# The role of mechanics in the growth and homeostasis of the intestinal crypt

**DOI:** 10.1007/s10237-020-01402-8

**Published:** 2020-11-21

**Authors:** A. A. Almet, H. M. Byrne, P. K. Maini, D. E. Moulton

**Affiliations:** 1 Wolfson Centre for Mathematical Biology, Mathematical Institute, University of Oxford, Andrew Wiles Building, Radcliffe Observatory Quarter, Woodstock Road, Oxford, OX2 6GG UK; 2 NSF-Simons Center for Multiscale Cell Fate Research, University of California, Irvine, Irvine, CA USA; 3 Department of Mathematics, University of California, Irvine, Irvine, CA USA

**Keywords:** Crypt, Homeostasis, Morphoelasticity, Elastic rod, Growth

## Abstract

We present a mechanical model of tissue homeostasis that is specialised to the intestinal crypt. Growth and deformation of the crypt, idealised as a line of cells on a substrate, are modelled using morphoelastic rod theory. Alternating between Lagrangian and Eulerian mechanical descriptions enables us to precisely characterise the dynamic nature of tissue homeostasis, whereby the proliferative structure and morphology are static in the Eulerian frame, but there is active migration of Lagrangian material points out of the crypt. Assuming mechanochemical growth, we identify the necessary conditions for homeostasis, reducing the full, time-dependent system to a static boundary value problem characterising a spatially heterogeneous “treadmilling” state. We extract essential features of crypt homeostasis, such as the morphology, the proliferative structure, the migration velocity, and the sloughing rate. We also derive closed-form solutions for growth and sloughing dynamics in homeostasis, and show that mechanochemical growth is sufficient to generate the observed proliferative structure of the crypt. Key to this is the concept of *threshold-dependent* mechanical feedback, that regulates an established Wnt signal for biochemical growth. Numerical solutions demonstrate the importance of crypt morphology on homeostatic growth, migration, and sloughing, and highlight the value of this framework as a foundation for studying the role of mechanics in homeostasis.

## Introduction

The crypts of Liehberkühn are a canonical example of biochemistry and biomechanics combining to maintain tissue homeostasis within a highly deformed morphology. These test-tube-shaped invaginations renew and maintain a protective epithelial layer, called the intestinal epithelium, for the small intestine and colon. In the context of disease, colonic cancer originates in the crypts (Humphries and Wright 2008), while during inflammation, crypts facilitate rapid regeneration of the epithelium (Seno et al. 2009). Therefore, proper crypt function is crucial to a healthy gut. Deciphering the numerous genetic and biochemical signalling pathways governing crypt homeostasis has been the focus of a significant amount of research. Mathematical and computational modelling has been particularly useful in providing insight. However, many aspects of crypt morphogenesis and homeostasis are still not well understood.

**Fig. 1:**
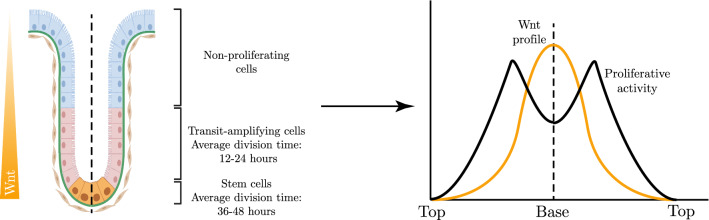
The internal proliferative structure of the crypt. The proliferative structure is bimodal as a function of position along the crypt, i.e. maximal between the crypt base and crypt top. However, Wnt signalling, thought to be the primary governor of proliferation, is unimodal, i.e. maximal at the base

One aspect of uncertainty concerns the unique and robust proliferative structure of the crypt. In the base of the crypt resides a pool of stem cells, which produce progenitors that migrate upwards. Transit-amplifying cells are the first progenitor cell type to emerge, proliferating rapidly for a fixed number of divisions as they migrate from the crypt base. Transit-amplifying cells differentiate into non-proliferating specialised cells, which reside at the top of the crypt. Despite the robustness of this hierarchical structure, it is not fully understood how it emerges. Wnt signalling is known to be a primary driver of proliferation within the crypt (Clevers 2006) and forms a decreasing spatial profile from the crypt base to the top (Gaspar and Fodde 2004). If proliferation within the crypt was driven solely by Wnt, then proliferative activity would be highest in the base with a monotonically decreasing profile moving towards the top; viewed as a function of arc length along a single crypt from top to top, we would observe a “unimodal” form of growth, peaking in the middle (the base). However, proliferative activity is concentrated in the transit-amplifying cell region, creating instead a “bimodal” growth profile that is maximal between the crypt base and the crypt edges (Alberts et al. (2002; Spit et al. 2018). This concept is illustrated in Fig. 1.

The second aspect of interest concerns homeostasis. Generically, homeostasis refers to a target state of a system, an equilibrium that is usually thought of as optimal in some way for the functioning of the system. In growing tissues, homeostasis is characterised by a balance between cell division and cell death or extrusion (Guillot and Lecuit 2013), such that the morphological properties of the tissue (shape, size) do not change with time. Homeostasis in the crypt is particularly interesting in this regard: the bimodal growth profile noted above is maintained during homeostasis, as is the deeply invaginated crypt morphology. This dynamic homeostasis requires a delicate balance of growth, cell migration, and the extrusion, or “sloughing”, of cells at the top of the crypt.

Numerous factors can contribute to growth regulation and the maintenance of a homeostatic state; these can be either chemical or physical. Our goal in this paper is to investigate the role of mechanics. Mechanical forces have been found to be a key contributor in growth regulation and in homeostasis in a number of systems (LeGoff and Lecuit 2016; Shraiman 2005). The principal idea in mechanically driven growth and homeostasis is that growth of a tissue depends on the difference between the stress in the tissue and a target homeostatic stress. For instance, parts of the tissue that are in relative tension compared to the target stress will grow to relieve the tension. In purely mechanical growth, mechanical homeostasis occurs when the stress is exactly equal to the target stress, at which point growth is halted (Taber 2009; Erlich et al. 2019). Mechanically driven growth may also be combined with other cues, (for example, biochemical) so that mechanical forces enhance or reduce the growth rate (Erlich et al. 2018). It is this latter case that is of interest here, with Wnt signalling acting as a well-known regulator of crypt proliferation (Spit et al. 2018).

In particular, we consider two questions:

1. Can growth driven by a unimodal biochemical signal (Wnt), but regulated by mechanical feedback, produce a “bimodal” proliferative structure?
2. What are the conditions on the system for dynamic homeostasis to be maintained, and can this be achieved consistently with (1)?

While these questions are motivated specifically by the crypt, similar questions may be relevant to a number of related systems. In a broader sense, the issues considered are: (i) whether mechanical feedback can qualitatively alter biochemical patterns of growth, and (ii) how to approach the mechanics of non-static tissue homeostasis. A key objective here is to formulate a modelling framework capable of treating such issues. While patterns generated solely through biochemical processes, otherwise known as Turing patterns, have been well studied and continue to be an active area of research (Crampin et al. 1999; Krause et al. 2020), we focus on mechanical pattern formation in this work, where the interplay between growth and stress drive the transition from a trivial, flat morphology to a non-trivial, buckled morphology.

Our approach will be to investigate these questions in a continuum setting. We model the cross-section of a single crypt, treating the line of epithelial cells as a growing, elastic rod. Similar models have appeared in the literature (Edwards and Chapman 2007; Nelson et al. 2011), in which the mechanics emerges from first principles and the resulting system is defined by partial differential equations, for which numerous computational and analytical tools are available. At the same time, considerable care must be taken when incorporating cellular-level processes, such as sloughing and localised growth, within a continuum framework. In terms of dynamic homeostasis, a subtle issue arises in even defining homeostasis, given that growth is not halted and, at the level of cells, there is a “treadmilling process”. Our starting point is to define homeostasis from a biologist’s perspective: an observer watching any particular point along the crypt would see a fixed rate of cell division and cell migration at all times, without any change in morphology. As we show, translating this concept to a precise definition in a continuum mechanics setting requires careful delineation of variables. While solid mechanics is most naturally expressed using Lagrangian variables, we show that a characterisation of dynamic homeostasis requires translation of the governing system to an Eulerian representation. In this way, we derive the necessary conditions relating growth rate, migration velocity, and sloughing rate for homeostasis to exist.

This paper is structured as follows. In Sect. 2, we present the mathematical framework for modelling dynamical tissue homeostasis that is of the type mentioned above. For a given form of mechano-chemical growth, we identify necessary conditions for homeostasis to occur in Sect. 3. In Sect. 4, we identify functional forms of mechanical feedback that generate the proliferative structure of the crypt, by considering the system in the absence of curvature. Returning to the original, 2D morphology, we compute the homeostatic states and analyse their dynamic stability in Sect. 5. We end this paper with a discussion of results and possible extensions in Sect. 6.

## Modelling framework

**Fig. 2:**
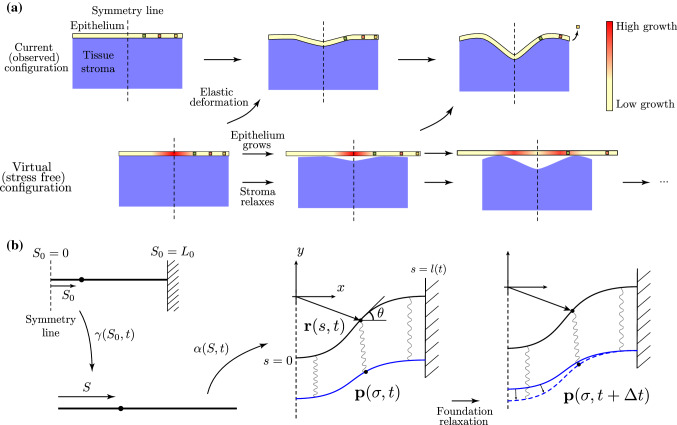
Model setup. **a** An idealisation of the developing crypt, comprising a growing epithelium and a passive tissue stroma. The stress free “virtual” configurations of the epithelium and stroma evolve due to (non-uniform) epithelial growth and stromal remodelling in response to the deformation. Lagrangian (material) points in the epithelium are marked by coloured squares. The non-uniform growth profile is indicated by the colour bar. **b** The mathematical model of the system consists of a growing planar elastic rod (the epithelium) that is attached to a foundation curve (the stroma) via a continuum of elastic springs. The deformation of the elastic rod is decomposed into a growth stretch and elastic stretch. Remodelling of the stroma is modelled by relaxation of the foundation curve. (In this schematic the springs are given a finite rest length; this is for visual purposes—in the model the rest length is taken to be zero)

We first outline the framework used to model the crypt. In its homeostatic state, the colonic crypt comprises a highly deformed morphology, to which various biochemical and biomechanical factors contribute. A biologically realistic description of the crypt must capture the interplay between chemical and mechanical cues, which both contribute to epithelial growth, as well as the migration and sloughing of material. We take a simplistic approach to biochemistry, modelling an ever-present Wnt signal profile. The mechanics is strongly linked to local and global constraints which include the basement membrane, to which the crypt is anchored, and the surrounding non-epithelial tissue stroma (Meran et al. 2017), as well as the geometric constraint imposed by neighbouring crypts. We exploit several modelling assumptions, outlined below and pictured schematically in Fig. 2a.

As the crypt shape is similar to that of a test tube, there is an approximate radial symmetry about the crypt base. Therefore, we can consider the crypt geometry from a cross-sectional view, as if one has taken a histological slice of the tissue, and we model only the transverse deformations. This allows for a convenient 1D parametrisation of the crypt epithelium, which we treat as a growing line of cells deforming within the $x-y$ plane. As the length of the crypt epithelium is much greater than the height and width of a single cell, and the depth of the crypt ($\sim 500\mu$m in homeostasis (Taylor et al. 2003)) is sufficiently long, we adopt a continuum approach, representing the proliferating line of cells as a growing, elastic rod embedded in a plane.

The supporting basement membrane and tissue stroma play a largely passive structural role in the crypt, providing resistance to any deformation of the epithelium. Remodelling is a feature commonly observed in tissue stroma (Bonnans et al. 2014), and seems particularly evident in the crypt, in which the stromal region is subjected to highly nonlinear morphological changes during the significant invagination that occurs during crypt morphogenesis (Bonnans et al. 2014; Hushka et al. 2020). Our view of the stroma/basement membrane (hereafter just called stroma) is thus as a passive elastic material that it is attached to the epithelium and relaxes its reference shape to the current shape of the crypt. Thus, at any given time, we can associate a stress free “virtual” configuration to both the epithelium and the stroma, which will evolve as the epithelium grows and the stroma relaxes (Fig. 2a).

Our mathematical description of this system consists of two physical components. The epithelium is modelled as an elastic rod, parameterised by the planar curve ${\mathbf {r}}=(x,y)$, that undergoes axial growth following the basic framework of morphoelastic rods (Moulton et al. 2013). In this description, the growth and elastic deformation of the rod are defined by three distinct configurations: an initial, pre-grown (Lagrangian) configuration, which is stress-free and parametrised by arc length $S_0$; a grown and stress-free configuration, parametrised by arc length *S*; and a current (Eulerian) configuration, parametrised by arc length *s*. The total lengths in the initial, grown, and current configurations are $L_0$, *L*, and *l*, respectively. The rod evolves via a 1D morphoelastic decomposition characterised by one-to-one maps between the different arc lengths (Fig. 2b): the growth stretch $\gamma = \partial S/\partial S_0$ maps Lagrangian points to the grown configuration, while the elastic stretch $\alpha = \partial s/\partial S$ describes the stretching or compressing of arc length from grown to current configuration. The total stretch $\lambda = \partial s/\partial S_0$ from initial to current arc length then satisfies

1 $$\begin{aligned} \lambda = \alpha \gamma \qquad \Longleftrightarrow \qquad \frac{\partial s}{\partial S_0} = \frac{\partial s}{\partial S}\frac{\partial S}{\partial S_0}. \end{aligned}$$

The second physical component is the non-epithelial stroma. Rather than model this region explicitly, we abstract the mechanical effects of this composite material into a single curve whose position generates a force density applied along the epithelial line. A similar abstraction has been employed in previous crypt models and related systems with growth on a substrate (Edwards and Chapman 2007; Nelson et al. 2011; Chirat et al. 2013). Explicitly, we define a foundation curve ${\mathbf {p}}(\sigma ,t)$ representing the shape and location of the stroma, and associate a one-to-one map between points on the rod and points on the foundation (more on this below); the resistive force of the stroma is then modelled by a force density applied to the rod that is a function of ${\mathbf {r}}-{\mathbf {p}}$ (taken here to be a linear function for simplicity). The remodelling of the stroma is then described by an evolution law for the foundation curve, such that the curve ${\mathbf {p}}$ relaxes to the shape of the rod, ${\mathbf {r}}$. Conceptually, we imagine a continuum of springs between the rod (epithelium) and foundation (stroma), such that the foundation relaxes to reduce the stress in the springs, as depicted in Fig. 2b.

To isolate a single crypt while still incorporating the geometric constraint imposed by neighbouring crypts, we consider a fixed horizontal domain in which the growing rod and foundation reside, and with a clamped boundary condition imposed for the rod. Since growth is presumed to persist even in homeostasis, this setup is only consistent with the loss of material at the boundary. This extrusion, or sloughing, forms a key feature of our modelling framework, and is illustrated schematically in Fig. 2a by the motion of Lagrangian material points towards the edge of the domain, where the orange point is ultimately extruded out of the crypt and thus removed from the computational domain. Our mathematical characterisation of this process is described further below.

In order to focus on mechanical effects, we take a simplistic approach to the biochemistry, assuming that there is a prescribed background concentration of Wnt present at each point along the rod. The growth of the rod is taken to be due to a combination of Wnt concentration and mechanical stress. We also assume that growth and remodelling occur on much slower timescales than that of any elastic deformations, so that the system is always in quasi-static mechanical equilibrium.

### Geometry and mechanics

We now outline the governing equations for the system. As growth is most naturally defined as a function of the initial arc length $S_0$, we first describe the governing equations with respect to $S_0$, though a reformulation to the Eulerian frame will be of principal importance for homeostasis (and note also that due to the nature of the one-to-one maps, any function can be written in terms of any of the arc length parameters $S_0$, *S*, or *s*).

The rod shape is described by its centreline, modelled as a 2D curve ${\mathbf {r}}(S_0, t) = x{\mathbf {e}}_x + y{\mathbf {e}}_y$. Let $\theta$ denote the angle between the tangent vector $\varvec{\tau }=\cos \theta {\mathbf {e}}_x + \sin \theta {\mathbf {e}}_y$ and the *x*-axis. Geometry supplies

2 $$\begin{aligned}&\frac{\partial x}{\partial S_0} = \alpha\gamma\cos\theta\end{aligned}$$

3 $$\begin{aligned}&\frac{\partial y}{\partial S_0} = \alpha \gamma \sin \theta . \end{aligned}$$

Note that the factor $\alpha \gamma$ arises when parametrising with respect to $S_0$.

Let ${\mathbf {n}}(S_0, t) = n_x{\mathbf {e}}_x + n_y{\mathbf {e}}_y$ denote the resultant force within the rod. The mechanical effects of the supporting basement membrane and tissue stroma are modelled through a foundation force proportional to $({\mathbf {r}}-{\mathbf {p}})$, where the 2D curve ${\mathbf {p}}(\sigma ,t) = p_x{\mathbf {e}}_x + p_y{\mathbf {e}}_y$ denotes the position of the foundation, where $\sigma$ parameterises material points of the foundation, but need not necessarily be arc length. This is similar to the classic Winkler foundation, except that here the foundation position evolves over time, such that the foundation relaxes to the current rod shape at rate $\eta ^{-1}$, which introduces a characteristic remodelling time scale $\eta$. That is, the foundation evolves via $\partial {\mathbf {p}}/\partial t=\eta ({\mathbf {r}}-{\mathbf {p}})$. From a mechanical perspective, some form of relaxation is crucial in generating realistic morphologies. In Appendix 1, we show the effect of a static foundation in crypt morphogenesis from flat to an invaginated state: with no relaxation, the resistive force at the crypt base continues to increase and a deeply invaginated state is never reached. For a more thorough investigation of alternative forms, such as a foundation with fixed position and viscoelastic springs, see Almet (2019).

The attachment between rod and foundation is defined through a one-to-one map between $\sigma$ and $S_0$. That is, we associate to each point along the rod a single point along the foundation, such that the forces and relaxation are defined point-wise through this one-to-one map. Explicitly, we can write $\sigma =H(S_0,t)$, where the “attachment function” *H* satisfies $H(0,t)=0$ and $\partial H/\partial S_0>0$; the expression $({\mathbf {r}}-{\mathbf {p}})$ then really means $({\mathbf {r}}(S_0,t)-{\mathbf {p}}(H(S_0,t),t)$^1^. We include the potential dependence on time in *H* to allow the attachments to evolve either due to growth or cell migration. In principle, this could be described with an additional constitutive law for the evolution of attachments, in which frictional force generation may also be incorporated. We do not pursue such details here, though we shall see that a natural attachment evolution emerges in the construction of the homeostatic state.

In component form, the balance of linear momentum and foundation relaxation read:

4 $$\begin{aligned}&\frac{\partial n_x}{\partial S_0} = \alpha \gamma Ek_f(x - p_x), \qquad \frac{\partial p_x}{\partial t}= \frac{1}{\eta }(x - p_x), \end{aligned}$$

5 $$\begin{aligned}&\frac{\partial n_y}{\partial S_0} = \alpha \gamma Ek_f(y - p_y), \qquad \frac{\partial p_y}{\partial t} = \frac{1}{\eta }(y - p_y). \end{aligned}$$

Here, *E* is the Young’s modulus of the rod, so that the dimensionless parameter $k_f$ relates foundation stiffness to rod stiffness. The factor $\alpha \gamma$ again appears because we express the system in Lagrangian form, so that the force density $Ek_f({\mathbf {r}}-{\mathbf {p}})$ is a force per current length.

Letting ${\mathbf {m}} = m{\mathbf {e}}_z$ denote the resultant rod moment, the balance of angular momentum is given by:

6 $$\begin{aligned}&\frac{\partial m}{\partial S_0} = \alpha \gamma (n_x\sin \theta - n_y\cos \theta ). \end{aligned}$$

The force and moment balance is supplemented by constitutive laws for bending and stretching. We relate the bending moment, *m*, to the flexure, $\partial \theta /\partial S$, through the standard relation:

7 $$\begin{aligned} m = \frac{EI}{\gamma }\frac{\partial \theta }{\partial S_0}, \end{aligned}$$

where *I* is the moment of inertia. Note that the moment is proportional to the flexure, $\partial \theta /\partial S$, rather than the curvature $\partial \theta /\partial s$, as $\partial \theta /\partial S$ is independent of any stretching within the rod. We also relate the elastic stretch $\alpha$ to the axial stress through a linear constitutive relation:

8 $$\begin{aligned}&n_\tau :={\mathbf {n}}\cdot \varvec{\tau }=n_x\cos \theta + n_y\sin \theta = EA(\alpha - 1), \end{aligned}$$

equivalent to a Hookean spring, where *A* is the area of the rod cross-section. The constitutive law (8) models the extensibility of the rod. Note that for an inextensible rod, (8) would be replaced by the geometric constraint $\alpha \equiv 1$.

Finally, the system is driven by imposing a growth law of the form:

9 $$\begin{aligned}&\frac{\partial \gamma }{\partial t}=\gamma \,G({\text {Wnt}},n_\tau , t, \dots ), \end{aligned}$$

where the function *G* could incorporate numerous effects, but in our analysis will depend only on Wnt concentration and axial stress $n_\tau$.

It remains to impose boundary and initial conditions. A typical crypt morphology is symmetric about the base. Supposing that the full crypt encompasses the region $-L_0\le x\le L_0$, here we exploit this symmetry and consider a half domain, valid for rod morphologies symmetric about $x = 0$ (see Fig. 2a). Thus, we restrict attention to the domain $x\in [0, L_0]$, shifting the point $S_0=0$ to the middle of the rod and imposing a symmetry condition at $S_0=0$ and a clamped boundary condition at $S_0=L_0$:

10 $$\begin{aligned}&x(0) = 0, \quad n_y(0) = 0, \quad \theta (0) = 0,\nonumber \\&x(L_0) = L_0, \quad y(L_0) = 0, \quad \theta (L_0) = 0. \end{aligned}$$

Natural initial conditions are a flat foundation and rod shape: $x(S_0,0)=p_x(S_0, 0) = S_0$ and $y(S_0, 0) =p_y(S_0, 0) = 0$, plus zero initial growth, $\gamma (S_0,0)=1$, in which case the force and moment are initially zero. While these conditions are needed in the context of morphogenesis, in our analysis of homeostasis we shall see that prescribing such conditions is not actually necessary.

### Non-dimensionalisation

To reduce the number of model parameters in the system, we non-dimensionalise independent and dependent variables in the following manner:

11 $$\begin{aligned} &t^*= T t,\nonumber \\ &(S_0^*, x^*, y^*, p_x^*, p_y^*)= L_0(S_0, x, y, p_x, p_y),\nonumber \\ &(n_x^*, n_y^*)= EIL_0^{-2}(n_x, n_y),\nonumber \\ &m^*= EIL_0^{-1}m, \end{aligned}$$

where *T* is the typical growth timescale. Substituting (11) into Eqs. (3)–(9) and dropping asterisks for notational convenience then yields the full non-dimensional system:

12 $$\begin{aligned} \frac{\partial x}{\partial S_0}&= \alpha \gamma \cos \theta , \end{aligned}$$

13 $$\begin{aligned} \frac{\partial y}{\partial S_0}&= \alpha \gamma \sin \theta , \end{aligned}$$

14 $$\begin{aligned} \frac{{\dot{\gamma }}}{\gamma }&=g= TG, \end{aligned}$$

15 $$\begin{aligned} \frac{\partial n_x}{\partial S_0}&=\alpha \gamma k(x - p_x), \qquad \dot{p}_x= \rho (x - p_x), \end{aligned}$$

16 $$\begin{aligned} \frac{\partial n_y}{\partial S_0}&= \alpha \gamma k(y - p_y), \qquad \dot{p}_y = \rho (y - p_y), \end{aligned}$$

17 $$\begin{aligned} \frac{\partial \theta }{\partial S_0}&= \gamma m, \end{aligned}$$

18 $$\begin{aligned} \frac{\partial m}{\partial S_0}&= \alpha \gamma (n_x\sin \theta - n_y\cos \theta ), \end{aligned}$$

19 $$\begin{aligned} n_\tau&= {\mathcal {S}}^{-1}(\alpha - 1), \end{aligned}$$

where *k* is the (non-dimensional) foundation stiffness and $\rho$ is the ratio of the growth timescale to the remodelling timescale of the foundation attachments. A larger value of *k* indicates a stiffer foundation, while larger values of $\rho$ correspond to more rapid relaxation of the foundation. Also, ${\mathcal {S}}$ is the “stretchability” of the rod, measuring the ratio of the bending stiffness to the stretching stiffness; the case ${\mathcal {S}} = 0$ corresponds to an inextensible rod (Pandey et al. 2014).

The boundary conditions (10) rescale to:

20 $$\begin{aligned}&x(0) = 0, \quad n_y(0) = 0, \quad \theta (0) = 0,\nonumber \\&x(1) = 1, \quad y(1) = 0, \quad \theta (1) = 0. \end{aligned}$$

The dimensionless Eqs. (12)–(19) contain three model parameters, *k*, $\rho$, and ${\mathcal {S}}$. Assuming a rectangular cross section with height *h* and width *w*, these parameters are given by:

21 $$\begin{aligned} k = \frac{12k_fL_0^4}{wh^3}, \qquad \rho = \frac{T}{\eta }, \qquad {\mathcal {S}} = \frac{h^2}{12L_0^2}. \end{aligned}$$

Based on estimates from human colonic crypt histologies (Taylor et al. 2003), we fix $w = 10\mu$m, $h = 15\mu$m, and $L_0 = 125\mu$m. We also set the growth timescale $T = 24$ hours to reflect the timescale of tissue morphogenesis. Later, we will fix $k_f = 0.01$ such that the crypt shape is even about $x = 0$ and contains a single invagination and fix $\rho = 10$, corresponding to rapid relaxation of the foundation to the rod shape, increasing the invagination depth (Almet 2019).

### Homeostasis: definition and framework

Before proceeding, it is important to define what is meant by homeostasis. As described in the Introduction, this will depend on the form of growth law, given by the function *g* in Eq. (14) . In the case of purely mechanical growth towards a homeostatic axial stress, $n_\tau ^*$, we would have $g=f(n_\tau -n_\tau ^*)$, where *f* is a function with the properties $f(0)=0$, $f(x)>0$ for $x>0$, and $f(x)<0$ for $x<0$, which models enhanced growth due to relative tension and the growth inhibition due to relative compression. In this case, homeostasis corresponds to the state with $n_\tau =n_\tau ^*$ for all $S_0$ and thus ${\dot{\gamma }}=0$ (as well as a static foundation). Such a state would truly be static, with all variables unchanging in time. More interesting, and relevant to the crypt, is a growth law that combines biochemical and biomechanical signals. Letting $W=W(S_0,t)$ denote the concentration of Wnt, we consider the generic additive form

22 $$\begin{aligned} g=W+f(n_\tau -n_\tau ^*). \end{aligned}$$

The choice of additive signals enables the biochemical and biomechanical components to be decoupled such that homeostasis requires a balance between chemistry and mechanics, i.e. $g=0\Leftrightarrow W=-f$.

Our baseline assumption is that the Wnt signal persists throughout time, and has a fixed functional form *in the Eulerian frame*. That is, Wnt can be viewed as a property of distance from the crypt base, described by the Eulerian variable *s*, as opposed to being a material property, described by the Lagrangian variable $S_0$. This reflects the notion that any biochemical process—for example, the diffusion of morphogens—is occurring in the current position of the tissue. Therefore, we prescribe a form $W=W(s)$.

**Fig. 3:**
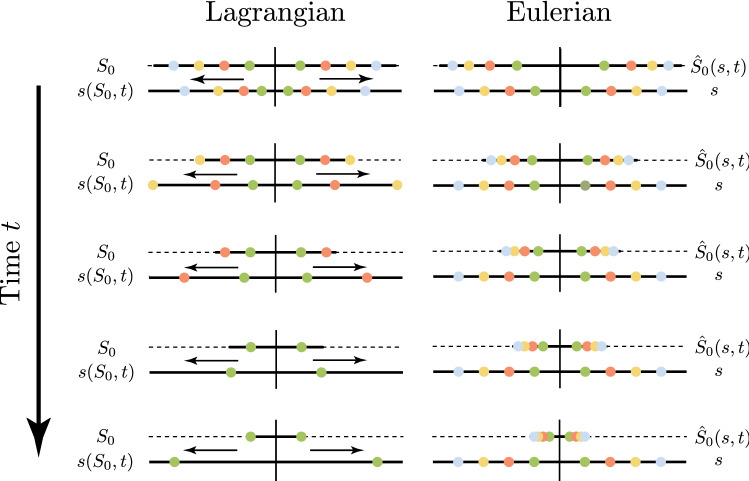
Demonstration of homeostasis in Lagrangian and Eulerian frames. The initial and current arc lengths are plotted for growth on a 1D line, simulating homeostasis in the crypt. Left column: the Lagrangian representation, with initial material arc length $S_0$ the independent variable. The marked circles correspond to $S_0 = 0.2$ (green), $S_0 = 0.4$ (orange), $S_0 = 0.6$ (yellow), and $S_0 = 0.8$ (blue) and their even extensions. Right column: the Eulerian representation, with current arc length *s* the independent variable, and circles at fixed $s = 0.2, 0.4, 0.6, 0.8$ (green, orange, yellow and blue circles, respectively) and even extensions. The evolution of the sloughing boundary $S_0 = L_\mu (t)$ is denoted by the dashed lines. In the Lagrangian configuration, material points move outwards until they are sloughed away and disappear; in the Eulerian configuration, the profile of *s* is fixed, and the map back to $S_0$ shows points converging to the centre as the $S_0$ domain shrinks

Given this, for growth to halt, the mechanosensitive term would need to cancel the Wnt signal exactly. In practice, however, homeostasis in the crypt is observed to be dynamic, a non-homogeneous treadmilling state, with growth persisting and balanced by sloughing of material at the top of the crypt. What *is* static in homeostasis is the growth rate and material velocity at any point *s*, as well as the morphology. In terms of experimental observations, a biologist watching a fixed point in space (in the Eulerian frame) will observe a constant rate of cell division and cell migration, and an unchanging crypt shape. Such a static state of growth and velocity in the Eulerian frame is *not static* in the Lagrangian frame. This concept is illustrated for a 1D geometry in Fig. 3, where we compare (in a simplified 1D morphology for illustration) a homeostatic process viewed in the Lagrangian and Eulerian frames. In the Eulerian view, with current arc length *s* as the independent variable, the current position (and foundation attachments) do not change with time, while the pre-image of each point, i.e. the material point $\hat{S}_0(s,t)$, continually moves inwards. By contrast, in the Lagrangian view, with material point $S_0$ as the independent variable, there is a clear migration outward of the current points $s(S_0, t)$ to the edge of the domain where they are ultimately removed from (sloughed out of) the Lagrangian domain. Thus, in homeostasis, while variables are fixed with respect to the Eulerian configuration, the Lagrangian configuration evolves continuously to maintain the homeostatic growth profile.

To express this notion of homeostasis mathematically, we first note that in the morphoelastic rods framework, growth rate is described by the incremental growth^2^${\dot{\gamma }}\gamma ^{-1}$, i.e. the function *g*, while the material velocity is $v=\partial {s}/\partial {t}$ and the morphology is fully determined by the function $\theta$. Homeostasis can then be defined by the condition that *g*, *v*, and $\theta$ are functions of *s* only (and not time *t*). To characterise homeostasis, it is thus prudent to cast the system in an Eulerian frame. In order to define quantities correctly, we must take particular care with time derivatives. To clarify the notation, we will use hats for a quantity expressed in Eulerian form. That is, for a function expressed in Lagrangian form $f(S_0, t)$, we denote its equivalent Eulerian form by $\hat{f}(s, t) := f(S_0(s, t), t)$. By the chain rule and the multiplicative decomposition (1), the associated space and time partial derivatives are:

23 $$\begin{aligned}&\frac{\partial \hat{f}}{\partial s} = \frac{\partial f}{\partial S_0}\frac{1}{\alpha \gamma }, \end{aligned}$$

24 $$\begin{aligned}&\frac{\partial \hat{f}}{\partial t} = \frac{\partial f}{\partial t} + \hat{v}\frac{\partial f}{\partial s}, \end{aligned}$$

where the (pull-back) velocity $\hat{v}(s, t)$ is given by

25 $$\begin{aligned} \hat{v}(s, t) = {\hat{\alpha }}{\hat{\gamma }}\frac{\partial \hat{S}_0}{\partial t}. \end{aligned}$$

The material velocity, $v(S_0, t) = \partial s(S_0, t)/\partial t$, measures the velocity at a fixed material point $S_0$—the continuum form of a cell migration velocity that is routinely measured in the crypt and provides a typical quantitative measure of homeostasis (Kaur and Potten 1986; Krndija et al. 2019). Applying the chain rule to $f(S_0, t) = \hat{f}(s(S_0,t), t)$ gives

26 $$\begin{aligned} \frac{\partial f}{\partial t} = \frac{\partial \hat{f}}{\partial t} + v\frac{\partial \hat{f}}{\partial s}. \end{aligned}$$

Rearranging (26) for $\partial \hat{f}/\partial t$ and equating this definition with Eq. (24) implies that the two velocities are related as follows:

27 $$\begin{aligned} v(S_0, t) = -\hat{v}(s(S_0, t), t). \end{aligned}$$

In the Eulerian frame, the spatial domain is $s \in [0, l]$, where $l=l(t)$ is the current rod length, satisfying

28 $$\begin{aligned} l= \int ^{1}_0\alpha \gamma dS_0. \end{aligned}$$

Following the conversions above, growth in the Eulerian configuration evolves according to the partial differential equation,

29 $$\begin{aligned} \frac{\partial {\hat{\gamma }}}{\partial t} = g(s,t){\hat{\gamma }} + \hat{v}\frac{\partial {\hat{\gamma }}}{\partial s}. \end{aligned}$$

From the multiplicative decomposition (1), we can compute the Eulerian form of the initial arc length $\hat{S}_0(s, t)$ via

30 $$\begin{aligned}&\frac{\partial \hat{S}_0}{\partial s} =\frac{1}{{\hat{\alpha }}{\hat{\gamma }}}. \end{aligned}$$

Applying (23)–(24) to the Lagrangian system (12)–(19), the remaining equations read:

31 $$\begin{aligned} \frac{\partial \hat{x}}{\partial s}&= \cos {\hat{\theta }}, \end{aligned}$$

32 $$\begin{aligned} \frac{\partial \hat{y}}{\partial s}&= \sin {\hat{\theta }}, \end{aligned}$$

33 $$\begin{aligned} \frac{\partial \hat{n}_x}{\partial s}&= k(\hat{x} - \hat{p}_x), \quad \frac{\partial \hat{p}_x}{\partial t} = \rho (\hat{x} - \hat{p}_x) + \hat{v}\frac{\partial \hat{p}_x}{\partial s}, \end{aligned}$$

34 $$\begin{aligned} \frac{\partial \hat{n}_y}{\partial s}&= k(\hat{y} - \hat{p}_y), \quad \frac{\partial \hat{p}_y}{\partial t} = \rho (\hat{y} - \hat{p}_y) + \hat{v}\frac{\partial \hat{p}_y}{\partial s}, \end{aligned}$$

35 $$\begin{aligned} \frac{\partial {\hat{\theta }}}{\partial s}&= \frac{\hat{m}}{{\hat{\alpha }}}, \end{aligned}$$

36 $$\begin{aligned} \frac{\partial \hat{m}}{\partial s}&= \hat{n}_x\sin {\hat{\theta }} - \hat{n}_y\cos {\hat{\theta }}, \end{aligned}$$

37 $$\begin{aligned} \hat{n}_\tau&= {\mathcal {S}}^{-1}\left( {\hat{\alpha }} - 1\right) , \end{aligned}$$

while the Eulerian boundary conditions take the form

38 $$\begin{aligned}&\hat{x}(0) = 0, \quad \hat{n}_y(0) = 0, \quad {\hat{\theta }}(0) = 0,\nonumber \\&\hat{x}(l) = 1, \quad \hat{y}(l) = 0, \quad {\hat{\theta }}(l) = 0. \end{aligned}$$

In the Eulerian view, we must again explicitly define the map between rod and foundation. In vector form, the foundation $\hat{\mathbf {p}}=\hat{\mathbf {p}}(\sigma ,t)$ is attached to the rod via an *Eulerian* attachment map ${\hat{\sigma }}=h(s,t)$. Given the Lagrangian map $\sigma =H(S_0,t)$, *h* would be defined via a composition of maps $h(s,t)=H(\hat{S}_0(s,t),t)$. Though as we shall see below, in the context of homeostasis a more natural approach is to define *h*, from which one can determine *H* similarly.

The Eulerian formulation requires particular care with two issues. One is that additional spatial derivatives have appeared, which must be balanced by extra boundary conditions. These relate to the velocity at the left boundary, $s = 0$, and are outlined below. The other issue concerns $\hat{S}_0(s, t)$. Observe that there are two first-order partial differential equations for $\hat{S}_0$, (25) and (30). To ensure that $\hat{S}_0$—and, consequently, the velocity $\hat{v}(s, t)$—are defined consistently, we require the following compatibility condition:

39 $$\begin{aligned} \frac{\partial ^2\hat{S}_0}{\partial s\partial t} = \frac{\partial ^2\hat{S}_0}{\partial t \partial s} \ \Longleftrightarrow \ \frac{\partial }{\partial s}\left( \frac{\hat{v}}{{\hat{\alpha }}{\hat{\gamma }}}\right) = \frac{\partial }{\partial t}\left( \frac{1}{{\hat{\alpha }}{\hat{\gamma }}}\right) , \end{aligned}$$

which connects the velocity to the growth and elastic stretch.

The Eulerian system as outlined above, including the compatibility equation, (39), holds in general, regardless of whether the system is in homeostasis or not. For most applications, it would be disadvantageous to solve the system in Eulerian form due to the changing spatial domain and extra derivatives and boundary conditions. For a dynamic homeostasis, however, the Eulerian formulation enables us to identify the conditions necessary for a homeostatic state.

Before addressing the specifics of homeostasis, the final step needed in our framework is a description of sloughing, i.e. the loss or extrusion of cells at the top of the crypt. If growth persists without changing the morphology, material must be lost. A loss of material at the boundary $s=l$ is thus equivalent to reducing the Lagrangian domain. To this end, we define the sloughing boundary

40 $$\begin{aligned} L_\mu (t) := \hat{S}_0(l, t) = \int ^l_0\frac{1}{{\hat{\alpha }}{\hat{\gamma }}}ds. \end{aligned}$$

At any time, the material domain is $\hat{S}_0\in [0,L_\mu (t)]$, such that the clamped boundary condition is applied at ${\hat{S}}_0=L_\mu$ instead of at ${\hat{S}}_0=1$, and thus the region $(L_\mu ,1]$ is effectively removed from the system. If, for instance, continued growth occurs (i.e. ${{\hat{\gamma }}}(s, t)$ is an increasing function of time) in an inextensible rod ($\hat{\alpha} \equiv 1$), and the current length *l* does not vary in time, (40) shows that the Lagrangian domain will shrink monotonically. With the definition of $L_\mu (t)$ from (40), which in itself is not particularly biologically meaningful, we will show in Sect. 3.1 how one can derive a more relevant sloughing *rate* that measures the net cell turnover rate.

**Fig. 4:**
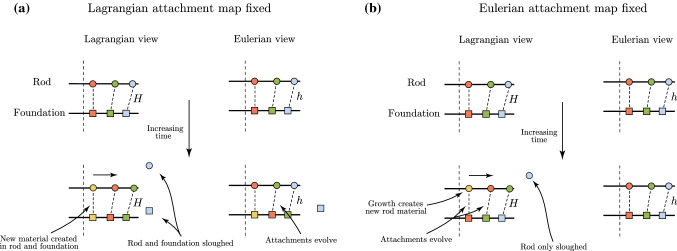
Evolving attachment maps in homeostasis. **a** If the Lagrangian attachment map is fixed, then both the rod and foundation are removed during sloughing and Eulerian attachments are dynamic. **b** A fixed Eulerian attachment map is reminiscent of the biologically realistic scenario: the foundation points remain fixed, while the material points of the rod migrate past the foundation and are sloughed away

Extrusion itself is an intricate mechanical process that can involve complex changes at the level of cell geometry (Eisenhoffer et al. 2012). In the crypt, it is only the epithelial cells that are extruded, not the substrate to which they are adhered (Williams et al. 2015). In our framework, since there is a one-to-one attachment between rod and foundation, extrusion introduces a subtle distinction in the Lagrangian versus Eulerian descriptions, and highlights the conceptual challenge in connecting the continuum description to the properties of a finite number of discrete cells. If the Lagrangian attachment map is fixed, i.e. $\sigma =H(S_0)$ is independent of time, then the material points of the substrate would also depart the computational domain, akin to stromal cells being extruded along with epithelial cells. The Eulerian attachment would then have to evolve to accommodate the “departing” substrate, see Fig. 4a. However, if the Eulerian attachment map remains fixed, i.e. $\sigma =h(s)$ is independent of time, then the material points of the foundation do not depart the computational domain, since the Eulerian domain does not shrink with sloughing. In this description, the *Lagrangian* attachment map will evolve, which corresponds physically to the material attachments updating continually as epithelial cells migrate out of the crypt. This second view, pictured schematically in Fig. 4b, is more consistent with the biological reality, and is the description we employ below. In particular, since $\sigma$ need not be an arc length parameter, we can choose $\sigma \equiv s$ in homeostasis without loss of generality; in this way, the attachment map would only appear when translating back to the evolution of Lagrangian attachments.

## Conditions for homeostasis

We now proceed to derive the necessary conditions for homeostasis. The starting point is the definition that incremental growth, morphology, and velocity, in Eulerian form, are all functions of *s* only. Thus, considering the mechanochemical incremental growth:

41 $$\begin{aligned} g(s,t) = W(s) + f(\hat{n}_\tau - \hat{n}^*), \end{aligned}$$

if $g=g(s)$ in homeostasis, it follows that ${\hat{n}}_\tau ={\hat{n}}_\tau (s)$. Since the shape is completely determined by ${{\hat{\theta }}}={{\hat{\theta }}}(s)$ in homeostasis, then by the definition ${\hat{n}}_\tau ={\hat{n}}_x\cos {{\hat{\theta }}}+{\hat{n}}_y\sin {{\hat{\theta }}}$, it must also be true that $\hat{n}_x$ and $\hat{n}_y$ are independent of time:

42 $$\begin{aligned} \hat{n}_\tau&= \hat{n}_\tau (s), \,{\hat{\theta }} = {\hat{\theta }}(s),\nonumber \\&\Longrightarrow \ \hat{n}_x = \hat{n}_x(s), \,\hat{n}_y = \hat{n}_y(s). \end{aligned}$$

By the constitutive relation (37), the Eulerian elastic stretch ${\hat{\alpha }}$ is also independent of time:

43 $$\begin{aligned} {\hat{\alpha }}(s) = 1 + {\mathcal {S}}\hat{n}_\tau (s). \end{aligned}$$

Next, note that $(\hat{x}, \hat{y})$ can be obtained straightforwardly from ${{\hat{\theta }}}(s)$:

44 $$\begin{aligned}&{\hat{x}}=\hat{x}(s) = \int ^s_0\cos {\hat{\theta }}(\xi )d\xi , \end{aligned}$$

45 $$\begin{aligned}&{\hat{y}}=\hat{y}(s) = \int ^s_0\sin {\hat{\theta }}(\xi )d\xi - \int ^l_0\sin {\hat{\theta }}(\xi )d\xi . \end{aligned}$$

Equations (33)–(34) then imply that $\hat{p}_x = \hat{p}_x(s)$ and $\hat{p}_y = \hat{p}_y(s)$. Furthermore, by Eqs. (36) and (42), the bending moment $\hat{m} = \hat{m}(s)$.

Setting the time derivatives, $\partial \hat{p}_x/\partial t$ and $\partial \hat{p}_y/\partial t$, to zero, we can then simplify the foundation relaxation equations to yield the following pair of first-order (in space) ordinary differential equations (ODEs):

46 $$\begin{aligned}&\hat{p}_x' = \frac{\rho (\hat{p}_x - \hat{x})}{\hat{v}}, \end{aligned}$$

47 $$\begin{aligned}&\hat{p}_y' = \frac{\rho (\hat{p}_y - \hat{y})}{\hat{v}}, \end{aligned}$$

where the prime $'$ denotes differentiation with respect to *s*. We can also obtain a first-order ODE for ${\hat{v}}(s)$ from the compatibility condition (39). Using $\partial {\hat{\alpha }}/\partial t = 0$, Eq. (39) simplifies to:

48 $$\begin{aligned} \hat{v}' = \frac{{\hat{\alpha }}'}{{{\hat{\alpha }}}}\hat{v} - g(s;{\hat{n}}_\tau ). \end{aligned}$$

Here, we write $g=g(s;{\hat{n}}_\tau )$ to make explicit that the axial stress profile is incorporated in the functional form of *g*.

To summarise, in homeostasis, the variables

49 $$\begin{aligned} {\mathbf {U}}=\{{\hat{x}},{\hat{y}},{{\hat{\theta }}},{\hat{n}}_x,{\hat{n}}_y, {{\hat{\alpha }}}, {\hat{p}}_x,{\hat{p}}_y,{\hat{m}},{\hat{v}}\} \end{aligned}$$

are all functions of *s* only. Note that this set does not include the growth ${{\hat{\gamma }}}$ nor the initial arc length ${\hat{S}}_0$, but this is to be expected: in dynamic homeostasis, growth persists and thus depends on time; it follows that the map from current to initial arc length does as well. Moreover, the set of first-order ODEs for ${\mathbf {U}}(s)$ decouple from ${{\hat{\gamma }}}$ and ${\hat{S}}_0$, which evolve in homeostasis according to:

50 $$\begin{aligned} \dot{{\hat{\gamma }}}&= g(s; \hat{n}_\tau (s)){\hat{\gamma }} + \hat{v}(s){\hat{\gamma }}', \end{aligned}$$

51 $$\begin{aligned} {{\hat{S}}_0}'&=\frac{1}{{{\hat{\alpha }}}(s){{\hat{\gamma }}}(s,t)}. \end{aligned}$$

How should one interpret the homeostatic system? The ODEs for ${\mathbf {U}}$ govern the relation between the incremental growth and velocity profiles (*g*(*s*) and ${\hat{v}}(s)$) and the Eulerian profiles for shape, force, moment, stretch, and foundation position. Equations (50)–(51) dictate how the growth and arc length maps must evolve in time to maintain those profiles. We solve this system computationally in Sect. 5.

Counting variables, there are ten spatial variables in ${\mathbf {U}}$, but as ${{\hat{\alpha }}}$ is known constitutively, there are only nine derivatives. At $s=0$, there are conditions on ${\hat{x}}$, ${{\hat{\theta }}}$, and ${\hat{n}}_y$, and conditions on ${\hat{x}}$, ${{\hat{\theta }}}$, and ${\hat{y}}$ at $s=l$, providing only six conditions. However, in the Eulerian frame there are “additional” spatial derivatives on ${\hat{v}}$, ${\hat{p}}_x$, and ${\hat{p}}_y$. By symmetry, the velocity at the centre must vanish, so $\hat{v}(0) = 0$. Consequently, from Eqs. (46)–(47), we must have that $\hat{p}_x(0) = \hat{x}(0) = 0$ and $\hat{p}_y(0) = \hat{y}(0)$, respectively, so that $\hat{p}_x'(s)$ and $\hat{p}_y'(s)$ are not singular at $s = 0$, thus giving an additional three conditions.

Therefore, in homeostasis, to obtain the spatial variables, ${\mathbf {U}}$, we solve Eqs. (31)–(36), and Eqs. (46)–(48), subject to the nine boundary conditions:

52 $$\begin{aligned}&\hat{x}(0) = 0, \quad \hat{n}_y(0) = 0, \quad {\hat{\theta }}(0) = 0,\nonumber \\&\hat{p}_x(0) = 0, \quad \hat{p}_y(0) = \hat{y}(0), \quad \hat{v}(0) = 0,\nonumber \\&\hat{x}(l) = 0, \quad \hat{y}(l) = 0,\quad {{\hat{\theta }}}(l) = 0. \end{aligned}$$

### Growth and sloughing dynamics in homeostasis

As shown, the time-independent variables ${\mathbf {U}}$ satisfy a boundary value problem (BVP) that can be solved independently from the variables ${{\hat{\gamma }}}$ and ${\hat{S}}_0$. Supposing such a solution has been found, we now construct a closed-form solution for ${{\hat{\gamma }}}$ and ${\hat{S}}_0$, and use this to identify the rate of sloughing at the boundary needed for a consistent dynamic homeostasis.

From the definition of ${\hat{v}}$ in Eq. (25), we see that in homeostasis, ${\hat{v}}(s){{\hat{\alpha }}}(s)^{-1}={{\hat{\gamma }}}(s,t)\partial \hat{S}_0(s,t)/\partial t$. Since the left-hand side is independent of *t*, the right-hand side must be as well, implying that $\partial \hat{S}_0/\partial t$ and ${\hat{\gamma }}$ decompose into separable functions of *s* and *t* with cancelling time components. In particular, we have:

53 $$\begin{aligned} \frac{\partial \hat{S}_0}{\partial t}= \frac{\varSigma (s)}{T(t)}, \qquad {\hat{\gamma }} = \varGamma (s)T(t). \end{aligned}$$

We first solve for ${\hat{\gamma }}$ by substituting the form (53) into Eq. (51). This yields the following:

54 $$\begin{aligned} \frac{\dot{T}}{T} = g(s) + \hat{v}(s)\frac{\varGamma '}{\varGamma } = \beta \in {\mathbb {R}}. \end{aligned}$$

Solving for *T* and $\varGamma$, respectively, yields the solutions

55 $$\begin{aligned}&T(t) = T_0{\mathrm {e}}^{\beta t}, \end{aligned}$$

56 $$\begin{aligned}&\varGamma (s) = \varGamma _0\ {\mathrm {exp}}\left( \int ^s_0\frac{\beta - g(s')}{\hat{v}(s')}ds'\right) . \end{aligned}$$

We note from the boundary condition $\hat{v}(0) = 0$ and (54) that $\beta$ is given by

57 $$\begin{aligned} \beta = g(0). \end{aligned}$$

Thus, we have an explicit form for growth in homeostasis:

58 $$\begin{aligned} {\hat{\gamma }}(s, t) = {\hat{\gamma }}_0\ {\mathrm {exp}}\left( g(0)t + \int ^s_0\frac{g(0) - g(s')}{\hat{v}(s')}ds'\right) , \end{aligned}$$

where ${\hat{\gamma }}_0 = \varGamma _0T_0$ is a constant.

Now, the solution (58) implies that $\partial \hat{S}_0/\partial t$ takes the form

59 $$\begin{aligned} \frac{\partial \hat{S}_0}{\partial t} = \varSigma (s){\mathrm {e}}^{-g(0)t}. \end{aligned}$$

Substituting (59) and (58) into Eq. (25) implies that

60 $$\begin{aligned} \varSigma (s) = -\frac{\hat{v}(s)}{g(0){\hat{\alpha }}(s)\varGamma (s)}. \end{aligned}$$

We substitute the solution (60) into the form (59) and integrate with respect to time, obtaining the general solution for $\hat{S}_0$:

61 $$\begin{aligned} \hat{S}_0(s, t)&= -\frac{\hat{v}(s){\mathrm {exp}}\left( -\int ^s_0\frac{g(0) - g(s')}{\hat{v}(s')}ds'\right) }{g(0){\hat{\gamma }}_0{\hat{\alpha }}(s)}{\mathrm {e}}^{-g(0)t}. \end{aligned}$$

We remark that the constant that arises by integrating (59) vanishes due to the boundary condition $\hat{S}_0(0, t) = 0$. Note also that since the foundation parameter $\sigma$ is chosen to satisfy $\sigma \equiv s$ in homeostasis, (61) also provides an expression for the evolving Lagrangian attachment map $\hat{S}_0(\sigma ,t)$ (this is the inverse of the map $\sigma =H(S_0,t)$, as outlined in Sect. 2.1). In particular, the exponential form implies that the rate of remodelling of attachments satisfies:

$$\begin{aligned} \frac{\partial \hat{S}_0(\sigma ,t)}{\partial t}=-g(0)\hat{S}_0(\sigma ,t). \end{aligned}$$

With our solutions to ${\hat{\gamma }}(s, t)$ and the homeostatic elastic stretch, ${\hat{\alpha }} = 1 + {\mathcal {S}}\hat{n}_\tau$, we can now calculate the sloughing boundary $L_\mu$ by evaluating (61) at $s = l$:

62 $$\begin{aligned} L_\mu (t) =-\frac{\hat{v}(l){\mathrm {exp}}\left( -\int ^l_0\frac{g(0) - g(s)}{\hat{v}(s)}ds\right) }{g(0){\hat{\gamma }}_0(1 + {\mathcal {S}}\hat{n}_\tau (l))}{\mathrm {e}}^{-g(0)t}. \end{aligned}$$

Therefore, in homeostasis, the sloughing boundary decays exponentially in time to balance the growth rate, with decay rate determined by *g*(0).

Note that the expression for $L_\mu (t)$ allows us to determine the, as-yet-unspecified constant, ${\hat{\gamma }}_0$. If we assume that homeostasis began at $t = 0$, then we have the initial condition $L_\mu (0) = 1$. In turn, this fixes the integration constant ${\hat{\gamma }}_0$ as:

63 $$\begin{aligned} {\hat{\gamma }}_0&= -\frac{\hat{v}(l)}{g(0)(1 + {\mathcal {S}}\hat{n}_\tau (l))}{\mathrm {exp}}\left( -\int ^l_0\frac{g(0) - g(s)}{\hat{v}(s)}ds\right) . \end{aligned}$$

Hence, the expression (62) simplifies significantly to

64 $$\begin{aligned} L_\mu (t) ={\mathrm {e}}^{-g(0)t}. \end{aligned}$$

To connect the sloughing boundary $L_\mu$ to a sloughing rate, denote by the quantity $\mu$ the total amount of material sloughed from the rod—physically this would be a measure of the total number of cells extruded from the crypt. This is equivalent to the amount of arc length in the grown configuration that is discounted by mapping $S_0=1$ to the right boundary. Thus, $\mu$ and $\gamma (S_0, t)$ are related by

65 $$\begin{aligned} \mu (t) = S(1) - S(L_\mu (t)) = \int ^1_{L_\mu (t)}\gamma dS_0. \end{aligned}$$

Differentiating (65) and applying Leibniz’s rule, we find that the sloughing rate, $\dot{\mu }$, relates to $\dot{L}_\mu$ via the integro-differential equation:

66 $$\begin{aligned} \dot{\mu } = -\dot{L}_\mu \gamma (L_\mu , t) +\int ^{1}_{L_\mu }\dot{\gamma } dS_0. \end{aligned}$$

By construction, no further growth occurs in the region past $S_0 = L_\mu$. Therefore, we set $\dot{\gamma } = 0$ in the region $S_0\in [L_\mu , 1]$. Furthermore, $\gamma (L_\mu , t) = {\hat{\gamma }}(l, t)$ and we can write

67 $$\begin{aligned} \dot{\mu } = -\dot{L}_\mu {\hat{\gamma }}(l, t). \end{aligned}$$

Differentiating (62) with respect to *t* and substituting the resulting expression into Eq. (67) yields the simplified quantity

68 $$\begin{aligned} \dot{\mu } = -\frac{\hat{v}(l)}{1 + {\mathcal {S}}\hat{n}_\tau (l)} = -\frac{\hat{v}(l)}{1 + {\mathcal {S}}\hat{n}_x(l)}, \end{aligned}$$

where we have used the right boundary condition (20) to note that $\hat{n}_\tau (l) = \hat{n}_x(l)$. Therefore, in homeostasis, while $L_\mu (t)$ decays exponentially at a rate determined by the incremental growth at the base, *g*(0), and the sloughing rate $\dot{\mu }$ is constant, with a value that depends on the migration velocity and the stress at the crypt top, $s = l$. Alternatively, the flow velocity Eq. (48) can be solved in terms of ${\hat{\alpha }}(s)$ and *g*(*s*), enabling us to express $\dot{\mu }$ as:

69 $$\begin{aligned} \dot{\mu } = \int ^l_0\frac{g(s)}{{\hat{\alpha }}(s)}ds. \end{aligned}$$

This expression provides an interpretation of the sloughing rate as balancing the net change of material arc length per unit time, given by integrating the incremental growth over the non-discarded portion of the domain.^3^ For instance, an increase in growth per unit time means more material must disappear at the boundary to maintain homeostasis.

## Form of mechanical feedback

Having formulated the homeostasis framework, we wish to analyse the structure of a homeostatic state. As the homeostatic system is nonlinear, constructing solutions will largely require numerical computation. To proceed, we must first prescribe the functional form of Wnt, *W*(*s*), and mechanical feedback, $f({\hat{n}}_\tau -n^*)$. For the Wnt signal, we consider a simple Gaussian form:

70 $$\begin{aligned} W(s) = \exp \left( -\frac{s^2}{\sigma _W^2}\right) , \end{aligned}$$

which has the desired monotonicity for $s > 0$ but also reflects the rapid decay of Wnt away from the base if the constant $\sigma _W < 1$(Marshman et al. 2002).

Less clear is a reasonable form of mechanical feedback, which is strongly linked to the first question posed in the Introduction: can mechanical feedback account for the bimodal growth profile, given a unimodal Wnt signal?

To gain insight, we first consider a simplified setting of 1D growth along a line. This will enable us to compare incremental growth profiles for different growth laws without having to account for differing 2D morphologies. Setting $\theta = 0$ and letting $n = n_x$ and $p = p_x$, the Lagrangian governing Eqs. (12)–(18) simplify to

71 $$\begin{aligned} \frac{\partial s}{\partial S_0}&= \alpha \gamma , \end{aligned}$$

72 $$\begin{aligned} \frac{\partial n}{\partial S_0}&= \alpha \gamma k(s - p), \quad \frac{\partial p}{\partial t} = \rho (s - p), \end{aligned}$$

where $\alpha$ relates to *n* through the 1D constitutive law

73 $$\begin{aligned} n = {\mathcal {S}}^{-1}(\alpha - 1), \end{aligned}$$

and the boundary conditions in a 1D geometry are:

74 $$\begin{aligned} s(0) = 0, \qquad s(1) = 1. \end{aligned}$$

We consider first a linear form of *f*, so that

75 $$\begin{aligned} \frac{\dot{\gamma }}{\gamma } = W(s) + \phi (n - n^*), \end{aligned}$$

where $\phi$ is a parameter describing sensitivity to mechanical stress, and thus the relative impact of mechanical feedback, and $n^* \le 0$ is the homeostatic stress. The law (75) models the continual regulation of Wnt signalling due to mechanical feedback. If $n- n^* > 0$, indicating relative tension, incremental growth $\dot{\gamma }\gamma ^{-1}$ is increased, while if $n - n^* < 0$, indicating relative compression, $\dot{\gamma }\gamma ^{-1}$ is decreased.

**Fig. 5:**
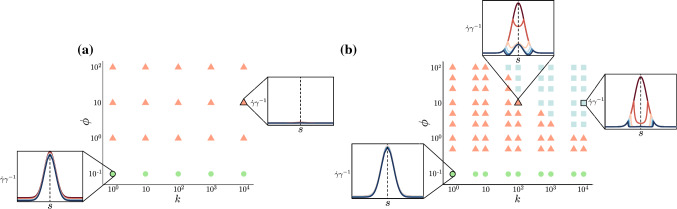
Threshold-based mechanical feedback generates a realistic crypt growth structure in 1D. We have set $\sigma = 0.24$, $\rho = 10$, and $n^* = -0.4$. For selected values of *k* and $\phi$, we plot the incremental growth profile, $\dot{\gamma }\gamma ^{-1}$, at times $t = 0, 1, 2, \dots , 5$. All plots of $\dot{\gamma }\gamma ^{-1}$ are to scale. Dark red lines correspond to earlier times, while dark blue lines correspond to later times. **a** Phase diagram of incremental growth structures for ever-present mechanical feedback (75). **b** Phase diagram of incremental growth structures for threshold-based mechanical feedback (76). Threshold-based mechanical feedback generates a richer phase space of growth profiles, including bimodal profiles as observed in the crypt

While the form of mechanical feedback considered in the growth law (75) is often used in studies of mechanical homeostasis (Erlich et al. 2019), there are two features of this “ever-present” feedback that may be interpreted as biologically unrealistic. First, we note that if the threshold stress $n^* < 0$, and the system begins at a stress-free state, $n\equiv 0$, then mechanical feedback instantly increases the growth. Second, and more importantly, the growth law (75) does not actually alter the monotonicity of the spatial profile of $\dot{\gamma }\gamma ^{-1}$. In the limiting case of an infinitely stiff foundation ($k\rightarrow \infty$), it can be proven (see Appendix 2) that the spatial profile of incremental growth will always have the same monotonicity as *W*. We show that this appears to hold more generally for finite values of *k* in Fig. 5 (discussed below).

Intuitively, we expect that in order for growth away from the base to overtake growth at the base, where the Wnt signal is highest, we would need the mechanical feedback to be triggered earlier at the base than at other points. As such, consider a variation where mechanical feedback is triggered only in relative compression:

76 $$\begin{aligned} \frac{\dot{\gamma }}{\gamma } = W(s) + \phi (n - n^*){\mathrm {H}}(n^* - n). \end{aligned}$$

Here, the Heaviside function ${\mathrm {H}}(x)$ effectively generates a spatially dependent delay of mechanical feedback, as it will now only occur at points $S_0$ for which $n(S_0) < n^*$.

There are now four parameters in the system, *k*, $\rho$, $\phi$, and $n^*$. Of these, simulations indicate that *k* and $\phi$ have the strongest qualitative effect on growth profile. The threshold stress $n^*$ has a limited range $-{\mathcal {S}}^{-1} < n^*$, imposed by the linear constitutive law and the lower bound on stretch, ${{\hat{\alpha }}}>0$, and, moreover, $n^*$ should be negative to avoid instantaneously triggering the mechanical feedback (since $n=0$ at $t=0$). The foundation relaxation parameter, $\rho$, only exacerbates or diminishes the effect of *k* on the spatial variation of *n*, so we do not consider its effect.

To understand the role of the feedback, we thus fix ${\mathcal {S}} = 1$, $\sigma = 0.24$, $\rho = 10$ and $n^* = -0.4$, and perform a parameter sweep over *k* and $\phi$. Equations (71)–(72) are solved numerically using the MATLAB package bvp4c. For each pair of $(k, \phi )$ values, we simulate Eqs. (71)–(72) subject to one of the growth laws (75) and (76) up to $t = 5$ and examine the resulting spatial profile of incremental growth, $\dot{\gamma }\gamma ^{-1}$, plotted as a function of *s* (and including the even extension of the profile to $s<0$). Fig. 5 summarises the results of the parameter sweep.

Figure 5a characterises the incremental growth in the case of the non-threshold-based growth law (75). For all parameter values, the resultant profiles are qualitatively the same, showing a monotonic shape with a peak in the middle, albeit with reduced amplitude due to the presence of mechanical feedback. For $(k, \phi ) = (10^4, 10)$, the amplitude reduction of ${\dot{\gamma }}\gamma ^{-1}$ is particularly evident. The monotonic shape is qualitatively the same as the imposed Wnt profile, thus, in this case the mechanical feedback is not sufficient to alter the point of maximal growth.

Figure 5b considers the threshold growth law (76). Here, the spatial behaviour of $\dot{\gamma }\gamma ^{-1}$ falls into one of three distinct parameter regimes:

1. **Primarily Wnt-driven:** Here, $\dot{\gamma }{\gamma }^{-1}$ is maximal at $s = 0$, and essentially follows $\dot{\gamma }\gamma ^{-1} = W(s)$. This occurs when mechanical feedback is insufficient ($\phi$ is too small) to dampen the effect of the Wnt gradient. We note that although our classification of behaviours is based on simulations run up to $t = 5$, the behaviour observed is independent of the simulation end time. We can be certain that this behaviour holds for longer simulation times since, as stated, the stress is bounded below, $n(S_0, t) > -{\mathcal {S}}^{-1}$, the inhibition of growth due to mechanical feedback is bounded and if $\phi$ is too small, then the contribution from mechanical feedback will be negligible.
2. **Non-monotonic from the base:** In this regime, $\dot{\gamma }\gamma ^{-1}$ is still maximal at $s = 0$, but mechanical feedback has reduced growth non-monotonically. There are even parameter regions where $\dot{\gamma }\gamma ^{-1} < 0$ for $0< s < 1$. This behaviour arises for the widest range of parameter values, as neither *k* nor $\phi$ are high enough to give rise to bimodal behaviour.
3. **Maximal away from the base and top:** In this case, $\dot{\gamma }\gamma ^{-1}$ no longer attains a local maximum at $s = 0$. As such, its even extension is bimodal. This occurs when mechanical stress, $n(S_0, t)$, exhibits sufficient spatial variation due to growth (*k* is sufficiently large) and mechanical feedback is sufficiently strong ($\phi$ is sufficiently large) such that mechanical inhibition occurs in the base first, before other regions are affected. If *k* is small, then (72) implies that the rod stress $n(S_0, t)$ is effectively constant. Contrastingly, if we take $k \rightarrow \infty$, then $n(S_0, t) = {\mathcal {S}}^{-1}(\gamma ^{-1} - 1)$, which is minimal where growth $\gamma$ is maximal, and vice versa.

This analysis demonstrates that mechanical feedback, together with unimodal biochemical signalling, can produce a bimodal form of incremental growth, if the feedback has threshold dependence and sufficiently high values of *k* and $\phi$. That is, given a background biochemical signal that decreases monotonically from the crypt base to the top, if growth is regulated by mechanical stress, but is only triggered at sufficient compression, then with strong feedback and high resistance to deformation from the underlying substrate, the growth profile can be qualitatively altered to no longer be monotonic. It remains to demonstrate that this result carries over to the 2D morphology; for this, we turn to solving the full homeostatic system.

## Computing homeostasis

To examine properties of homeostasis, in this section, we solve the homeostatic BVP for the spatial variables ${\mathbf {U}}$ numerically, extract the growth profile and sloughing rate, and examine the properties and dependence on model parameters. We will also analyse the *dynamic stability* of the homeostatic solutions, which relates to the robustness of the homeostasis profiles while maintaining quasi-static equilibrium, and discuss how this compares to the more classical *inertial stability*.

**Fig. 6:**
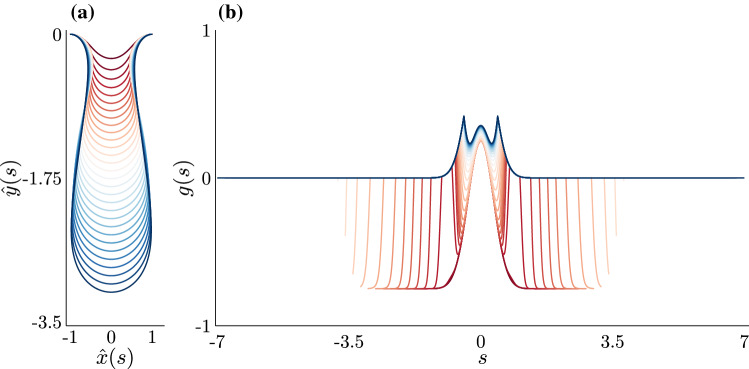
Computed homeostatic solutions for different values of total rod length, *l* . Solutions to Eqs. (31)–(39) are obtained from numerical continuation in *l* over the values $l = 1.2, 1.4, \dots , 6.8, 7$. Dark red lines indicate solutions for smaller values of *l*, while dark blue lines indicate solutions for larger values of *l*. The chosen model parameter values are $k = 868.056\ (k_f = 0.01)$, $\rho = 10$, $\phi = 0.75$, and $\hat{n}_\tau ^* = -3$. **a** The homeostatic morphologies $(\hat{x}(s), \hat{y}(s))$ for increasing *l*. Each solution has been reflected about $x = 0$ to better represent possible crypt morphologies. **b** The resulting homeostatic incremental growth profiles *g*(*s*) for increasing *l*, which have been reflected about $s = 0$ to better reflect possible proliferative structures of the crypt. For larger values of *l*, the spatial structure of *g*(*s*) resembles the crypt’s proliferative structure, demonstrating the link between morphology and homeostatic growth

In the Eulerian framework, note that the total arc length, *l*, is effectively a free parameter; that is, for any given *l*, one could seek a solution to the BVP. If we model the current arc length to be only slightly larger than the initial domain length, i.e. $l=1+\epsilon$ for $\epsilon \ll 1$, then the crypt shape will be nearly flat. In this limit, we can thus determine a solution as an asymptotic expansion about the state ${\hat{\theta} \equiv 0}$. Similar to a buckling analysis, infinitely many solutions exist, with increasing mode, yet only one will be stable in the classical sense. Having computed the stable mode, we then perform a numerical continuation with increasing *l*, thus producing more crypt-like homeostatic profiles with deeper invaginations.

### Examining the homeostatic solutions

For 2D morphologies, we consider an altered form of threshold-dependent mechanical feedback:

77 $$\begin{aligned} g(s) = W(s) + \phi \tanh (\hat{n}_\tau - \hat{n}_\tau ^*){\mathrm {H}}(\hat{n}_\tau ^* - \hat{n}_\tau ), \end{aligned}$$

where the effect of mechanical feedback saturates. Not only is this form of mechanical feedback more physically realistic, but it is more amenable to numerical continuation in *l*. For the form of mechanical feedback specified by (76), setting $\phi$ large enough to generate both the desired homeostatic morphology and, consequently, the correct incremental growth profile for larger values of *l* can result in numerical singularities for smaller values of *l* if $g(0) \le 0$. As we demonstrate in Appendix 2, the altered form (77) has the same qualitative features as (76) in the 1D geometry.

For given *l*, the time-independent variables ${\mathbf {U}}$ are found as solutions to the Eulerian system (31)–(39), using the Eulerian boundary conditions (52). These were computed numerically using a shooting method implemented with the Mathematica package NDSolve. We set the foundation stiffness parameter, $k = 868.056$, corresponding to a foundation stiffness scaling of $k_f = 0.01$, generating a mode one instability upon buckling; the foundation relaxation parameter to $\rho = 10$, corresponding to a rapidly relaxing foundation that contributes to a deeply invaginated morphology; the width of the Gaussian Wnt profile to $\sigma _W = 0.24$; the mechanical feedback strength to $\phi = 0.75$ in order to sufficiently alter the monotonicity of the homeostatic incremental growth $g(s; \hat{n}_\tau )$ for larger values of *l*; and the threshold axial stress to $\hat{n}_\tau ^* = - 3$ so that mechanical feedback inhibits growth in the crypt base ($s = 0$) but not at the crypt top ($s = l$).

In Fig. 6, we plot the homeostatic morphologies, $(\hat{x}(s), \hat{y}(s))$, and resultant homeostatic incremental growth profiles, *g*(*s*), that were obtained from numerical continuation in *l*—each colour corresponds to a value of *l* increasing from $l=1.2$ (red) to $l=7$ (blue). Unsurprisingly, in Fig. 6a, as the total rod length increases, the rod morphology becomes increasingly invaginated, a known feature of post-buckled elastic rods (Edwards and Chapman 2007; Nelson et al. 2011; Almet et al. 2019; Chirat et al. 2013). Figure 6b shows that the incremental growth profile also varies with *l*. For smaller values of *l*, where the morphologies exhibit little invagination, the growth profile is in fact non-negative only around $s = 0$, indicating that the homeostatic profile at small lengths can only be maintained with a resorption of material over most of the domain. Interestingly, we observe a qualitative transition in the growth profile, so that at larger values of *l*, when the morphology is significantly invaginated, the incremental growth profiles are both non-negative everywhere and also maximal away from the base, resembling the bimodal proliferative structure of the crypt. This confirms the intuition drawn from the 1D geometry in Sect. 4 on producing bimodal growth through mechanical feedback, while the qualitative dependence on *l* highlights the non-trivial relationship between morphology and growth structure.

**Fig. 7:**
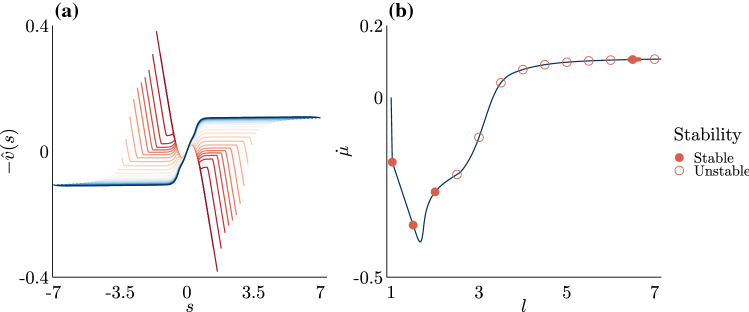
Biologically relevant measurements from the homeostatic solutions. **a** The migration velocity, defined by $-\hat{v}(s)$. Migration velocity solutions are obtained from numerical continuation of Eqs. (31)–(39) in *l*, over the range $l = 1.2, 1.4, \dots , 6.8, 7$, for assigned model parameter values of $k = 868.056\ (k_f = 0.01)$, $\rho = 10$, $\phi = 0.75$, and $\hat{n}_\tau ^* = -3$. Dark red lines correspond to solutions for smaller values of *l*, while dark blue lines correspond to solutions for larger values of *l*. In line with the emergence of realistic *g*(*s*) profiles, physically realistic migration velocity profiles only begin to emerge for larger values of *l*. **b** The sloughing rates $\dot{\mu }$ for various values of *l*, which balance incremental growth to dynamic tissue homeostasis. We have marked the dynamic stability of several solutions along this curve at $l = 1.025, 1.5, 2, \dots , 6.5, 7$. We have also highlighted the region $l \in [6.4, 6.7)$ to indicate that all computed solutions in this region are stable

In addition to the homeostatic growth structure, in Fig. 7, we plot the migration velocity profile $-\hat{v}(s)$ and sloughing rate ${\dot{\mu }}$ for increasing length *l*. These parameters form continuum versions of experimentally accessible measurements for the crypt, and with significant biological relevance, as they provide insight into the health status of the epithelium, where efficient migration and turnover are important for maintaining the intestinal epithelium (Potten et al. 1997; Sansom et al. 2004). Fig. 7a shows that for smaller values of *l*, when there is little crypt invagination, the migration velocity is negative over much of the domain (for $s>0$), akin to cells migrating *down* into the crypt, towards the base; though the velocity remains positive near the base, implying a stagnation point. It is only at larger values of *l* that the velocity attains a more realistic profile, where it represents cells migrating purely outwards. In particular, there is an approximately linear increase in velocity from $s = 0$, corresponding to the region of growth $g(s)>0$, and a flat velocity in the outer part of the domain where $g(s)\approx 0$. This feature is consistent with experimental observations that report a linear increase in migration velocity from the crypt base (Kaur and Potten 1986); Krndija et al. (2019).

For each length *l*, the sloughing rate ${\dot{\mu }}$ required to maintain the homeostatic state is plotted in Fig. 7b. Note that a positive value of ${\dot{\mu }}$ corresponds to a net loss of material (extrusion of cells), while ${\dot{\mu }}<0$ would require the unphysical addition of material at the boundary. We observe that the sloughing is only positive for larger *l*, when the morphology is sufficiently invaginated. In particular, note that for larger values of *l*, $\dot{\mu } \approx 0.1$, which corresponds to a net turnover time in the crypt of $\dot{\mu }^{-1}$ which, in dimensional units, corresponds to $T\dot{\mu }^{-1} \approx 10$ days and on the order of turnover time scales observed *in vivo* (Gehart and Clevers 2018).

We emphasise that the solutions presented in Figs. 6 and 7, obtained from numerical continuation in *l*, are not to be interpreted as a time sequence; that is, they do not represent a transition from development to homeostasis. Rather, homeostasis is *imposed* at each length, and time is a hidden parameter at each length through which total growth ${{\hat{\gamma }}}$ and initial arc-length ${\hat{S}}_0$ continue to evolve.

The quantities depicted in Figs. 6 and 7 reflect a delicate balance between growth, stress, and morphology in homeostasis. These plots also present two qualitatively distinct regimes: homeostasis at small *l* is characterised by very little invagination and a monotonic growth profile with resorption and a downwards migration of material over much of the domain. This state consists of material lost from negative incremental growth that can only be maintained by “negative” sloughing at the boundary, which adds material at the boundary to balance negative growth. These characteristics are all highly unphysical for the crypt. On the other hand, at larger *l* the picture is completely different: here, the homeostatic morphology has a deep invagination, bimodal growth profile with positive (or zero) growth throughout and an outward migration of material, where the net gain of material is maintained by positive sloughing at the boundary. Each of these features is consistent with observations in the crypt. Indeed, the differences between the two regimes may provide some insight as to why the crypt has such a deeply invaginated morphology in homeostasis, or rather, why homeostasis is not established until the crypt is deeply invaginated. The characteristics needed for homeostasis at small *l*, in particular negative growth and sloughing, are not physically possible in this model, so the system cannot remain in such a state.

### Dynamic stability analysis

An important feature of the intestinal crypt, and of biological homeostasis in general, is its robustness over time and the ability of the system to return to homeostasis after perturbation due to, for example, injury or genetic mutation. Having computed the structure of typical homeostatic solutions and demonstrated that properties of the crypt can be replicated within our continuum framework, we now investigate the robustness of these homeostatic states to perturbations. In other words, we are concerned with the notion of *dynamic* stability: whether a homeostatic state is recovered following a perturbation to the system variables. Here, we restrict to small dynamic perturbations, as they are amenable to a linear stability analysis.

Note that dynamical stability is not mechanical stability in the classical sense of incorporating inertia within the momentum balance equations. Rather, the question is whether the system returns to the homeostatic state if the variables are perturbed on the slow timescale of growth (Erlich et al. 2019). To answer this question, we perform a linear stability analysis on the quasi-static system^4^. Here, we must take care to perturb the system in a consistent way, given that there are two time-dependent variables, $\hat{S}_0(s, t)$ and ${\hat{\gamma }}(s, t)$, while the other dependent variables, summarised in (49), are functions of *s* only.

We perturb the time-independent variables as follows:

78 $$\begin{aligned} {\hat{\nu }}(s) = {\hat{\nu }}^{(0)}(s) + \varepsilon {\hat{\nu }}^{(1)}(s){\mathrm {e}}^{\xi t}, \end{aligned}$$

where ${\hat{\nu }}(s) \in {\mathbf {U}}$; $\varepsilon \ll 1$ is an arbitrarily small parameter; and the sign of $\xi \in {\mathbb {R}}$ determines the growth or decay of the perturbation and, consequently, the stability of the homeostatic solutions. Here and below, the superscript (0) refers to the homeostatic profile, such as those obtained in Sect. 5.1, while superscript (1) denotes the perturbed spatial profile, which must be determined as part of the stability analysis.

In Sect. 3.1, we showed that $\hat{S}_0(s, t)$ and ${\hat{\gamma }}(s, t)$ decay and grow, respectively, on a timescale determined by *g*(0), the homeostatic incremental growth at the base. In order to expand the time-dependent variables in a manner that is consistent with the time-independent variables, we write:

79 $$\begin{aligned}&\hat{S}_0(s, t) = \varSigma ^{(0)}(s){\mathrm {e}}^{-\beta t} + \varepsilon \varSigma ^{(1)}(s){\mathrm {e}}^{-\beta t}{\mathrm {e}}^{\xi t}, \end{aligned}$$

80 $$\begin{aligned}&{\hat{\gamma }}(s, t) = {\hat{\gamma }}_0\varGamma ^{(0)}(s){\mathrm {e}}^{\beta t} + \varepsilon {\hat{\gamma }}_0\varGamma ^{(1)}(s){\mathrm {e}}^{\beta t}{\mathrm {e}}^{\xi t}, \end{aligned}$$

where $\beta = g^{(0)}(0)$. The system at *O*(1) is satisfied by the homeostatic solution. At $O(\varepsilon )$, we obtain a linearised BVP with eigenvalue $\xi$. We have solved this by implementing a determinant method that involves integrating multiple linearly independent copies of the system from $s=0$ to $s=l$; the details are presented in Appendix 4. For the homeostatic solution to be dynamically stable, all eigenvalues $\xi$ must satisfy ${\mathrm {Re}}(\xi ) < 0$. If there is at least one eigenvalue ${\mathrm {Re}}(\xi ) > 0$, then the solution is dynamically unstable.

We have computed the stability of homeostatic solutions for various values of *l* in Fig. 7, and have labelled the stability in Fig. 7b. We find that for smaller *l*, up to $l = 2$, the homeostatic state is stable. As *l* increases, a transition to unstable homeostatic states is observed. A transition back to stable homeostatic states then occurs for a small region around $l = 6.5$; all solutions in the region $l \in [6.4, 6.7)$ are computed to be stable. While there may be other stable solutions that exist for $l > 7$, we could not access these solutions due to difficulties with numerical continuation. Also of note is that we find (results not plotted) that higher buckling modes, which are inertially unstable, are also dynamically stable for small values of *l*, a feature that highlights the distinction between dynamic and inertial stability.

As stated before, it is only at larger *l* that the homeostatic state resembles the characteristics of the crypt. While we have only presented results for a small subset of parameter space, and it would be imprudent to reach draw strong of conclusions in the context of the crypt, it is nevertheless interesting that we have uncovered a small region of homeostatic states that have both physically realistic characteristics and are dynamically stable. How a biological system regulates its size and *selects* homeostasis is one of the fundamental open questions in biology (Goriely 2016); while our model is undoubtedly too simplified to answer this question for the crypt, the ideas presented here may provide new insight as well as a new tool for analysis.

## Discussion

In this paper, we developed a mathematical model of tissue homeostasis in the intestinal crypt. The model built on morphoelastic rod theory, a continuum mechanics framework. Modelling tissue homeostasis as a dynamic process required careful translation of the mechanical description from the typical Lagrangian frame to the Eulerian frame, where the concept of tissue homeostasis is most naturally defined. A continuum framework enabled us to investigate the role of mechanics, both in generating the proliferative structure of the crypt, and in maintaining this structure in homeostasis.

A key starting point for our framework was a clear definition of homeostasis. Here, we translated the biologist’s view—a spatially heterogeneous treadmilling state with constant growth and velocity at each point in a laboratory frame—to our setting, and this produced a clear statement of certain system variables being time-independent when expressed in an Eulerian frame. In doing so, we demonstrated how the same quantities in the Lagrangian reference frame must evolve over time to maintain a static Eulerian frame, resulting in a migration of (Lagrangian) material points out of the crypt, not unlike the conveyor belt mechanism observed *in vivo* (Krndija et al. 2019). Correspondingly, the removal of material in the form of sloughing emerges naturally to balance growth in homeostasis. A clear advantage of the continuum framework was that it enabled closed-form solutions for both growth and the sloughing rate to be constructed.

In order to simulate growth and homeostasis in the crypt, we required explicit assumptions about the key contributors to growth. Here, we assumed mechanochemical growth, focussing on the role of mechanical (axial) stress in regulating the well-known Wnt signal profile that is present along the crypt. We showed that threshold-dependent mechanical feedback, where mechanical inhibition of growth is only triggered at points that are sufficiently compressed, can generate the observed growth structure of the crypt. This is not unlike the contact inhibition model that is considered in individual-based crypt models (Osborne et al. 2017). In contrast, the commonly used “ever-present” mechanical feedback law was incapable of generating the correct growth structure.

In homeostasis, the time-independent variables in the Eulerian system decoupled from the time-dependent variables, allowing the homeostatic state to be fully resolved by solving a spatial BVP. Therefore, we were able to compute homeostatic states using numerical continuation, much like standard buckling problems. Here, the continuation parameter was the total rod length in the homeostatic state. Numerical solutions revealed how the homeostatic incremental growth structure, migration velocity, and sloughing rate depends on the morphology (and stress profiles), suggesting that a significantly invaginated morphology may actually be necessary for crypt homeostasis, as plausible homeostatic growth and velocity profiles and sloughing rates emerged only for deep invaginations. However, dynamic stability analysis, which provided insight into the “robustness” of the constructed homeostatic solutions, revealed that many of these homeostatic states that generated the correct growth structure were dynamically unstable, despite being inertially stable, i.e. the preferred buckling mode. From an experimental point of view, this type of instability would correspond to a perturbation of the homeostatic state (say through injury or a small change in growth rate) causing a significant change in growth structure and/or morphology.

Extensions of the work presented here may naturally proceed in two distinct directions: specialisation to the crypt, and generalisation to explore more broadly the role of mechanics in homeostasis. With regard to the former, there are a number of ways to specialise our framework to that of the crypt. One assumption we have made is that all material parameters are spatially and temporally homogeneous. Viewing points along the rod as representative of a growing line of cells along the crypt is akin to the assumption that all cells have the same physical properties and response to biochemical signalling at all times. Spatial heterogeneity was only included in the Wnt signal profile, which consequently induced a heterogeneous response to mechanical feedback. It would be straightforward to include spatial or temporal dependence on other system properties, though of course one may have to trade analytical tractability for physical accuracy.

Also of note is that mechanical feedback was modelled as a phenomenological process that occurs instantaneously when triggered. However, it is known that each crypt contains a diverse population of cell types with varying mechanical properties and responses to chemical signals (such as Wnt) (Spit et al. 2018). In particular, it has been shown that YAP and TAZ, known mechanotransduction pathways, regulate the cellular response to Wnt signalling due to mechanical stress (Azzolin et al. 2014). At the cellular scale, Wnt is regulated by YAP and TAZ shuttling between the cell nucleus and cell cytoplasm, depending on mechanical stress. The shuttling mechanism means that there is a certain time lag between the activation of mechanical feedback and the resultant inhibition of Wnt. In other words, the response to mechanical feedback, modelled through the parameter $\phi$, may in fact be time dependent. Connecting model parameters, such as the Wnt response, *W*(*s*), and mechanical feedback strength, $\phi$, to more detailed mathematical models of biochemical signalling pathways, such as Wnt and YAP/TAZ, which can be more readily perturbed and tested in wet lab experiments, would allow us to validate and refine the role of mechanics in the crypt.

In order to keep the biochemistry as simple as possible, here we modelled Wnt as the sole biochemical regulator of proliferative capacity and investigated possible ways that Wnt may be regulated by mechanical stress. By modelling mechanochemical growth, we showed that it is possible to generate the proliferative structure of the crypt through this minimal growth law. Additionally, the assumption of mechanochemical growth increased the analytical tractability of the homeostasis framework considerably. However, there are numerous signalling pathways involved in crypt homeostasis (Spit et al. 2018). For example, BMP signalling has been established to negatively regulate proliferation in the crypt by driving terminal differentiation of stem cells (Haramis et al. 2004). As such, it may be possible to generate the same proliferative structure through purely biochemical processes, say, through the interaction of Wnt and BMP signalling.

In the other direction, separate from the crypt, a broader goal of this paper was to formulate a continuum framework that links growth and mechanics in dynamic homeostasis. Here, we have uncovered a number of interesting features and challenges that may naturally be explored in more detail and perhaps in a more generic setting. At a broad mechanical level, the system consists of a thin growing layer on a substrate, a scenario observed in many different contexts. Our particular modelling framework for the substrate as an evolving foundation stands in contrast to the common continuum approach of the reduction of an elastic half-space, e.g. (Brau et al. 2010; Budday et al. 2015). Our choice allows for a computationally efficient description of nonlinear deformations on a finite domain and with substrate relaxation, but it is not based on a rigorous reduction that starts from considering the properties of the full tissue and thus may not accurately capture all elastic or viscoelastic responses, e.g. shear. Connecting such distinct approaches more explicitly, both in continuum and discrete models, forms an important step in unifying the mechanical understanding of such systems. In our “rod on evolving foundation” model, we have also outlined a description of evolving attachments between the two layers, and shown the subtle but important distinctions in an Eulerian versus Lagrangian description. In this first instance, we have opted for the simplest model of these attachments, and have not explicitly included a constitutive law for the evolving attachments and the forces that may be generated therein. Incorporating such details will be an important extension and may enable, more generally, a new approach to continuum modelling of cell migration (Chen et al. 2020).

There exists an intriguing connection between the homeostatic sloughing we defined at the boundary and studies that have modelled surface growth as an evolving reference configuration (Zurlo and Truskinovsky 2017, 2018; Truskinovsky and Zurlo 2019). While we have studied a 1D rod structure, both for simplicity and as a reasonable idealisation of the crypt, in principle, the ideas we have presented for constructing homeostasis in an Eulerian frame could carry over to 2D surfaces and 3D bodies, though almost certainly with added complications. Also of interest are several works (Tomassetti et al. 2016; Abi-Akl et al. 2019; Abi-Akl and Cohen 2020; Abeyaratne et al. 2020) that have considered how surface growth can lead to “treadmilling” and the notion of a “universal growth path”, a concept that is connected to the developmental trajectory towards homeostasis, a feature that is absent from our study. Computing such trajectories within our modelling framework would certainly provide valuable insight, though it is not clear how our framework would have to be adapted to do so. While this forms an appealing avenue of future work, the nonlinear behaviour and rich solution structure we have uncovered in the case of imposed homeostasis highlights the significant challenge in providing an answer to the fundamental question of how growth is determined and regulated in biology.

## Figures and Tables

**Fig. 8:**
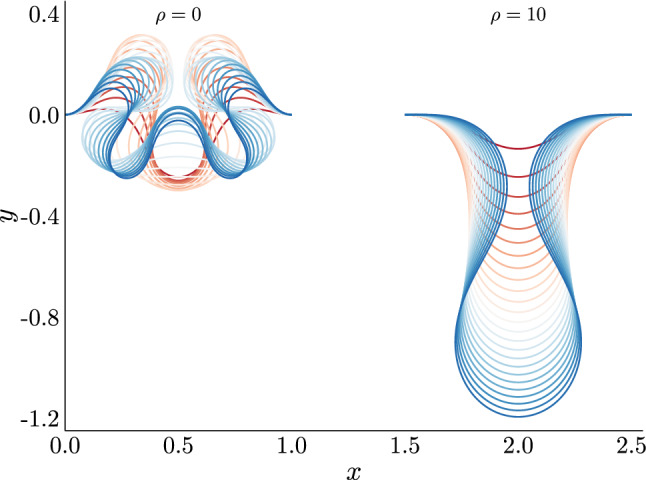
Allowing foundation attachment remodelling generates a more significant crypt invagination. For a purely static elastic foundation, corresponding to $\rho = 0$, increasing growth eventually results in an increase in mode number. Setting $\rho = 10$, so that the foundation attachments remodel sufficiently fast, allows the rod to maintain a realistic crypt morphology for increasing growth

**Fig. 9:**
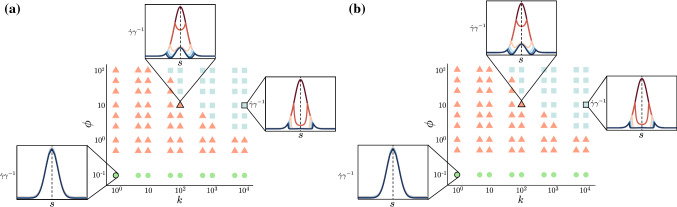
Saturating threshold-dependent mechanical feedback is indistinguishable from linear threshold-dependent mechanical feedback in a 1D geometry. **a** The phase space of growth profiles for linear threshold-dependent mechanical feedback (76), as shown in Fig. 5b. **b** The phase diagram for saturating threshold-dependent mechanical feedback (91). The phase diagrams and representative incremental growth profiles are identical in a 1D geometry

## Appendix 1: Introducing foundation remodelling generates a more realistic crypt morphology

We demonstrate that incorporating a foundation remodelling law allows continued rod invagination, which generates a more realistic crypt morphology than the static elastic foundation. A more thorough investigation of the different effects of the foundation constitutive laws on rod morphology can be found in Almet (2019).

We consider an actively deforming rod during, for example, morphogenesis, which can be appropriate described by the Lagrangian system, Eqs. (12)–(18). For simplicity, we consider constant, spatially homogeneous growth, $\gamma = 1 + t$, where $t > 0$ is the growth time, and assume an inextensible rod, which corresponds to setting the elastic stretch $\alpha \equiv 1$. Note that this means that the constitutive law (19) no longer applies. Following Sect. 5, we set the foundation stiffness parameter $k = 868.056$, corresponding to a foundation stiffness scaling of $k_f = 0.01$. We compare the static elastic foundation to a rapidly remodelling foundation. To consider a static foundation, we set the remodelling parameter, $\rho$, in Eqs. (15)–(16), to $\rho = 0$. To consider a foundation that remodels sufficiently fast, we set $\rho = 10$.

We obtain numerical solutions for the static foundation case using the numerical continuation package AUTO-07p (Doedel et al. 2007) and for the remodelling foundation case using the MATLAB package bvp4c. The results of the numerical continuations are shown in Fig. 8. The differences in morphologies are clear. When the rod evolves on a static foundation, the magnitude of the resistive force continues to increase. As growth continues, a larger penalty on rod invagination is imposed by the increase in foundation energy. Eventually, the foundation force causes the rod to fission, leading to an increase in mode number. In contrast, when the foundation attachments remodel to the current rod position, the resistive force does not increase as significantly over time and, in fact, decreases in magnitude. Over the same growth times, the rod attached to a remodelling foundation can continue invaginating and generate a deeper invagination than the static foundation case.

As evidence from the literature suggests that the crypt begins as a mode one instability and maintains this buckling mode throughout morphogenesis (Sumigray et al. 2018), it is clear that a sufficiently remodelling foundation allows us to generate a realistic crypt morphology. We assume that the same mechanical principles that govern crypt formation also hold for homeostasis, and thus retain this foundation law when deriving the necessary conditions for homeostasis.

## Appendix 2: Ever-present mechanical feedback does not disrupt a monotonic Wnt signal

Here, we prove that in the limiting case of an infinitely stiff foundation, $k \rightarrow \infty$, on a 1D geometry, the type of ever-present mechanical feedback modelled in (75),

$$\begin{aligned} \frac{\dot{\gamma }}{\gamma } = W(s) + \phi (n - n^*), \end{aligned}$$

does not alter the monotonicity of the Wnt signal, *W*(*s*), and thus the monotonicity of incremental growth, $\dot{\gamma }\gamma ^{-1}$. We deduce that for an infinitely stiff foundation, ever-present mechanical feedback will not generate a bimodal proliferative structure. Combined with the results from Fig. 5a, which are for a finite foundation stiffness, we conclude that this simple form of ever-present mechanical feedback is insufficient for replicating the proliferative structure of the crypt. While it is more difficult to prove that more complex forms of ever-present mechanical feedback cannot generate bimodal proliferative structures, we conjecture that threshold-dependent mechanical feedback is necessary to generate a bimodal proliferative structure, as well as being a more biologically realistic form of mechanical feedback.

In the limiting case of an infinitely stiff foundation, $k \rightarrow \infty$, we can solve Eqs. (71)–(74) analytically and obtain solutions for the current arc length, $s(S_0, t)$, and stress, $n(S_0, t)$:

81 $$\begin{aligned} s(S_0, t) = S_0, \qquad n(S_0, t) = \frac{1 - \gamma }{{\mathcal {S}}\gamma }. \end{aligned}$$

Substitution of the solution (81) into the growth law (75) yields a first-order, linear ODE for $\gamma (S_0, t)$:

82 $$\begin{aligned} \dot{\gamma } = \gamma W(S_0) + \phi \left( {\mathcal {S}}^{-1}(1 - \gamma ) - n^*\gamma \right) . \end{aligned}$$

Recalling the initial condition $\gamma (S_0, 0) = 1$, we deduce the following expressions for $\gamma$ and $\dot{\gamma }\gamma ^{-1}$:

83 $$\begin{aligned} \gamma (S_0, t)&= \frac{A_0 + A_1{\mathrm {e}}^{-gt}}{g}, \end{aligned}$$

84 $$\begin{aligned} \frac{\dot{\gamma }}{\gamma }&= \frac{-A_1g{\mathrm {e}}^{-gt}}{A_0 + A_1{\mathrm {e}}^{-gt}} \end{aligned}$$

where $A_0$, $A_1$, and *g* are given by:

85 $$\begin{aligned} A_0&= \phi {\mathcal {S}}^{-1},\nonumber \\ A_1&= \phi n^* - W(S_0),\nonumber \\ g&= \phi ({\mathcal {S}}^{-1} + n^*) - W(S_0). \end{aligned}$$

Note that $A_0$, $A_1$, and *g* are all functions of $W(S_0)$. With the closed-form expression (84), we can now make the following claim.

### Claim

Let $\dot{\gamma }\gamma ^{-1}$ evolve according to (84). Assume that $W(S_0)$ is a monotonically decreasing function of $S_0$. Then, $\dot{\gamma }\gamma ^{-1}$ is also a monotonically decreasing function of $S_0$.

### *Proof*

We must show that for $S_1 \ge S_2$, the following inequality is satisfied for all time $t > 0$:

86 $$\begin{aligned} \frac{\dot{\gamma }}{\gamma }\Big |_{S_0 = S_1} \ge \frac{\dot{\gamma }}{\gamma }\Big |_{S_0 = S_2}. \end{aligned}$$

Denote $w_1 := W(S_1)$ and $w_2 := W(S_2)$, respectively. Substituting (85) into expression (84), we must show:

87 $$\begin{aligned}&\frac{(w_1 - \phi n^*)(\phi ({\mathcal {S}}^{-1} + n^*) - w_1){\mathrm {e}}^{-(\phi ({\mathcal {S}}^{-1} + n^*) - w_1)t}}{\phi {\mathcal {S}}^{-1} + (\phi n^* - w_1)){\mathrm {e}}^{-(\phi ({\mathcal {S}}^{-1} + n^*) - w_1)t}}\nonumber \\&\ge \frac{(w_2 - \phi n^*)(\phi ({\mathcal {S}}^{-1} + n^*) - w_2){\mathrm {e}}^{-(\phi ({\mathcal {S}}^{-1} + n^*) - w_2)t}}{\phi {\mathcal {S}}^{-1} + (\phi n^* - w_2){\mathrm {e}}^{-(\phi ({\mathcal {S}}^{-1} + n^*) - w_2)t}}. \end{aligned}$$

By our assumption, $w_1 \ge w_2$. It then follows that $w_2 - \phi n^* \ge w_1 - \phi n^*$ and ${\mathrm {e}}^{-(\phi ({\mathcal {S}}^{-1} + n^*) - w_2)t} \ge {\mathrm {e}}^{-(\phi ({\mathcal {S}}^{-1} + n^*) - w_1)t}$. Therefore, we may bound the right-hand side of the inequality (87) above by:

88 $$\begin{aligned}&\frac{(w_2 - \phi n^*)(\phi ({\mathcal {S}}^{-1} + n^*) - w_2){\mathrm {e}}^{-(\phi ({\mathcal {S}}^{-1} + n^*) - w_2)t}}{\phi {\mathcal {S}}^{-1} + (\phi n^* - w_2){\mathrm {e}}^{-(\phi ({\mathcal {S}}^{-1} + n^*) - w_2)t}}.\nonumber \\&\le \frac{(w_1- \phi n^*)(\phi ({\mathcal {S}}^{-1} + n^*) - w_2){\mathrm {e}}^{-(\phi ({\mathcal {S}}^{-1} + n^*) - w_2)t}}{\phi {\mathcal {S}}^{-1} + (\phi n^* - w_1){\mathrm {e}}^{-(\phi ({\mathcal {S}}^{-1} + n^*) - w_1)t}}. \end{aligned}$$

Therefore, to prove that the inequality (87) holds, it suffices to show that:

89 $$\begin{aligned}&(\phi ({\mathcal {S}}^{-1} + n^*) - w_2){\mathrm {e}}^{-(\phi ({\mathcal {S}}^{-1} + n^*) - w_2)t}\nonumber \\&\le (\phi ({\mathcal {S}}^{-1} + n^*) - w_1){\mathrm {e}}^{-(\phi ({\mathcal {S}}^{-1} + n^*) - w_1)t}. \end{aligned}$$

Equivalently, we may simplify (89) and show:

90 $$\begin{aligned} {\mathrm {e}}^{(w_1 - w_2)t} \ge \frac{w_2 - \phi ({\mathcal {S}}^{-1} + n^*)}{w_1 - \phi ({\mathcal {S}}^{-1} + n^*)}. \end{aligned}$$

Observe that, as $w_1 \ge w_2$, the right-hand side of (90) is bounded above by 1. Moreover, as $t > 0$, it must also be true that ${\mathrm {e}}^{(w_1 - w_2)t} \ge 1$ for all $t > 0$. Therefore, the inequality (90) and, consequently, the inequality (86), hold for all time $t > 0$. That is, for a monotonically decreasing Wnt signal profile, the resultant incremental growth for the ever-present mechanical feedback law (75) is also monotonically decreasing function of $S_0$. Hence, on an infinitely stiff foundation, ever-present mechanical feedback is insufficient to generate the correct proliferative structure.

## Appendix 3: Saturating threshold-dependent mechanical feedback in 1D

In this section, we present numerical solutions of the 1D system (71)–(74). We show that, in the absence of morphology, saturating threshold-dependent mechanical feedback, modelled by the following law:

91 $$\begin{aligned} \frac{\dot{\gamma }}{\gamma } = W(s) + \phi \tanh (n - n^*){\mathrm {H}}(n^* - n), \end{aligned}$$

does not produce significantly different behaviours from the linear threshold-dependent mechanical feedback, which is modelled by the growth law (76):

$$\begin{aligned} \frac{\dot{\gamma }}{\gamma } = W(s) + \phi (n - n^*){\mathrm {H}}(n^* - n). \end{aligned}$$

As in Sect. 4, we perform a sweep over the foundation stiffness, *k*, and the strength of mechanical feedback, $\phi$. We classify the resulting incremental growth profiles, $\dot{\gamma }\gamma ^{-1}$, according to three different cases: primarily Wnt-driven, where $\dot{\gamma }\gamma ^{-1} \approx W(s)$; non-monotonic from the base, where $\dot{\gamma }\gamma ^{-1}$ is maximal at $s = 0$, but does not generate the desired proliferative structure; and maximal away from the base and top, where $\dot{\gamma }\gamma ^{-1}$ resembles the proliferative structure of the crypt. The remaining model parameters are set to $\rho = 10$, ${\mathcal {S}} = 1$, $\sigma _W = 0.24$, and $n^* = -0.4$. The respective phase diagrams for linear and saturating threshold-dependent mechanical feedback are plotted in Fig. 9. Over the considered values of *k* and $\phi$, the results are indistinguishable. Not only does the classification of the profiles of $\dot{\gamma }\gamma ^{-1}$ split into the same distinct parameter regions, but the representative profiles of $\dot{\gamma }\gamma ^{-1}$ are identical. Therefore, in a 1D geometry, linear threshold-dependent mechanical feedback is sufficient to generate the crypt’s proliferative structure. However, in 2D, the rod stretchability satisfies ${\mathcal {S}} \ll 1$ for a realistic crypt geometry, resulting in a much wider range of stress values than those observed in 1D. Consequently, the effect of mechanical feedback for a linear threshold-dependent law is amplified significantly. To render the computation of homeostatic solutions more amenable to numerical solution, we adopt the 2D equivalent of the growth law (91), where the 1D stress, *n*, is replaced by the axial stress, $n_\tau$, in (77). This law is arguably a more physically realistic mechanism for mechanical feedback, as it is likely that the effect of mechanical feedback does not increase indefinitely.

## Appendix 4: Stability analysis

We describe the technical details of the procedure that we use to compute the dynamical stability of the homeostatic solutions, given by the set of time-independent solutions, ${\mathbf {U}}$, and the time-dependent solutions, ${\hat{\gamma }}(s, t)$ and $\hat{S}_0(s, t)$. However, we note that we do not need to explicitly consider the stability of $\hat{S}_0(s, t)$, as it is embedded in the definition of the flow velocity, $\hat{v}(s)$, through (25).

As stated in Sect. 5.2, for each time-independent solution ${\hat{\nu }}(s) \in {\mathbf {U}}$, we expand the variables as follows:

92 $$\begin{aligned} {\hat{\nu }}(s)&= {\hat{\nu }}^{(0)}(s) + \varepsilon {\hat{\nu }}^{(1)}(s){\mathrm {e}}^{\sigma t}, \end{aligned}$$

93 $$\begin{aligned} {\hat{\gamma }}(s,t)&= {\hat{\gamma }}_0{\hat{\varGamma }}^{(0)}(s){\mathrm {e}}^{\beta t} + \varepsilon {\hat{\gamma }}_0{\hat{\varGamma }}^{(1)}(s){\mathrm {e}}^{\beta t}{\mathrm {e}}^{\sigma t}, \end{aligned}$$

where $\beta = g^{(0)}(0)$, the homeostatic incremental growth at the crypt base. At *O*(1), we recover the homeostatic system outlined in Sect. 3. At $O(\varepsilon )$, we obtain the following linearised boundary value problem:

94 $$\begin{aligned}&\hat{x}^{(1)'} = -{\hat{\theta }}^{(1)}\sin {\hat{\theta }}^{(0)}, \end{aligned}$$

95 $$\begin{aligned}&\hat{y}^{(1)'} = {\hat{\theta }}^{(1)}\cos {\hat{\theta }}^{(0)}, \end{aligned}$$

96 $$\begin{aligned}&\hat{n}_x^{(1)'} = k(\hat{x}^{(1)} - \hat{p}^{(1)}_x), \end{aligned}$$

97 $$\begin{aligned}&\hat{n}_y^{(1)'} = k(\hat{y}^{(1)} - \hat{p}^{(1)}_y), \end{aligned}$$

98 $$\begin{aligned}&\hat{p}_x^{(1)'} = \frac{(\sigma +\rho )\hat{p}^{(1)}_x - \rho \hat{x}^{(1)}}{\hat{v}^{(0)}}\nonumber \\&\qquad \quad + \frac{\rho (\hat{x}^{(0)} - \hat{p}_x^{(0)})}{(\hat{v}^{(0)})^2}\hat{v}^{(1)}, \end{aligned}$$

99 $$\begin{aligned}&\hat{p}_y^{(1)'} = \frac{(\sigma + \rho )\hat{p}^{(1)}_y -\rho \hat{y}^{(1)}}{\hat{v}^{(0)}}\nonumber \\&\qquad \quad + \frac{\rho (\hat{y}^{(0)} - \hat{p}_y^{(0)})}{(\hat{v}^{(0)})^2}\hat{v}^{(1)}, \end{aligned}$$

100 $$\begin{aligned}&{\hat{\theta }}^{(1)'} = \frac{\hat{m}^{(1)}}{{\hat{\alpha }}^{(0)}} - \frac{\hat{m}^{(0)}}{\left( {\hat{\alpha }}^{(0)}\right) ^2}{\hat{\alpha }}^{(1)}, \end{aligned}$$

101 $$\begin{aligned}&\hat{m}^{(1)'} = \hat{n}_x^{(1)}\sin \theta ^{(0)} - \hat{n}_y^{(1)}\cos \theta ^{(0)}\nonumber \\&\qquad \quad + \theta ^{(1)}(\hat{n}_x^{(0)}\cos \theta ^{(0)} + \hat{n}_y^{(0)}\sin \theta ^{(0)}), \end{aligned}$$

102 $$\begin{aligned}&\hat{v}^{(1)'} = \frac{{\hat{\alpha }}^{(0)'}}{\alpha ^{(0)}}\hat{v}^{(1)} + \frac{\hat{v}^{(0)}}{{\hat{\alpha }}^{(0)}}{\hat{\alpha }}^{(1)'}- \phi f'\left( \hat{n}^{(0)}_\tau - \hat{n}^*_\tau \right) \hat{n}^{(1)}_\tau \nonumber \\&\qquad \quad - \frac{\left( {\hat{\alpha }}^{(0)'}\hat{v}^{(0)} + \sigma {\hat{\alpha }}^{(0)}\right) }{\left( {\hat{\alpha }}^{(0)}\right) ^2}{\hat{\alpha }}^{(1)}, \end{aligned}$$

103 $$\begin{aligned}&\varGamma ^{(1)'} = \frac{(\beta + \sigma - W(s))}{\hat{v}^{(0)}}\varGamma ^{(1)} - \frac{\varGamma ^{(0)'}}{\hat{v}^{(0)}}\hat{v}^{(1)}\nonumber \\&\qquad \quad - \frac{\phi f'\left( \hat{n}^{(0)}_\tau - \hat{n}^*_\tau \right) \varGamma ^{(0)}}{\hat{v}^{(0)}}\hat{n}^{(1)}_\tau , \end{aligned}$$

where ${\hat{\alpha }}^{(1)}$ is found by expanding the constitutive relation (37):

104 $$\begin{aligned} {\hat{\alpha }}^{(1)}&= {\mathcal {S}}\Big (\hat{n}_x^{(1)}\cos {\hat{\theta }}^{(0)} + \hat{n}_y^{(1)}\sin {\hat{\theta }}^{(0)}\nonumber \\&\qquad \quad + {\hat{\theta }}^{(1)}\left( \hat{n}_y^{(0)}\cos {\hat{\theta }}^{(0)} - \hat{n}_x^{(0)}\sin {\hat{\theta }}^{(0)}\right) \Big ). \end{aligned}$$

Consequently, the derivative ${\hat{\alpha }}^{(1)'}$ can be determined.

The linearised boundary conditions are found by applying the Eulerian boundary conditions (38) and the velocity boundary condition $\hat{v}(0) = 0$, yielding the left boundary conditions:

105 $$\begin{aligned}&\hat{x}^{(1)}(0) = 0, \end{aligned}$$

106 $$\begin{aligned}&\hat{n}_y^{(1)}(0) = 0, \end{aligned}$$

107 $$\begin{aligned}&{\hat{\theta }}^{(1)}(0) = 0, \end{aligned}$$

108 $$\begin{aligned}&\hat{v}^{(1)}(0) = 0, \end{aligned}$$

109 $$\begin{aligned}&\hat{p}_x^{(1)}(0) = 0, \end{aligned}$$

110 $$\begin{aligned}&\hat{p}_y^{(1)}(0)= \frac{\rho }{\sigma + \rho }\hat{y}^{(1)}(0), \end{aligned}$$

111 $$\begin{aligned}&\varGamma ^{(1)}(0) = \frac{\phi f'(\hat{n}_x^{(0)}(0) - \hat{n}_\tau ^*)}{\sigma }\hat{n}_x^{(1)}(0), \end{aligned}$$

and the right boundary conditions:

112 $$\begin{aligned}&\hat{x}^{(1)}(l) = 0, \end{aligned}$$

113 $$\begin{aligned}&\hat{y}^{(1)}(l) = 0, \end{aligned}$$

114 $$\begin{aligned}&{\hat{\theta }}^{(1)}(l) = 0. \end{aligned}$$

We note that the linearised boundary value problem can be expressed in the compact form, $\hat{\mathbf {x}}^{(1)'} = {\mathbf {A}}\hat{\mathbf {x}}^{(1)}$, where $\hat{\mathbf {x}}^{(1)}$ is the linearised solution vector

$$\begin{aligned} \hat{\mathbf {x}}^{(1)} =\left( \hat{x}^{(1)},\hat{y}^{(1)},\hat{n}_x^{(1)},\hat{n}^{(1)}_y,\hat{p}^{(1)}_x,\hat{p}^{(1)}_y,{\hat{\theta }}^{(1)},\hat{m}^{(1)},\hat{v}^{(1)},\varGamma ^{(1)}\right) ^T, \end{aligned}$$

and ${\mathbf {A}}$ is the coefficient matrix and a function of both *s* and $\sigma$. Furthermore, we observe from (105)–(111) that there are no known left boundary conditions for $\hat{y}^{(1)}$, $\hat{n}_x^{(1)}$ and $\hat{m}^{(1)}$. To overcome this, we write $\hat{\mathbf {x}}^{(1)}$ as three linearly independent copies:

115 $$\begin{aligned} \hat{\mathbf {x}}^{(1)} = c_1\hat{\mathbf {x}}_1^{(1)} + c_2\hat{\mathbf {x}}_2^{(1)} + c_3\hat{\mathbf {x}}_3^{(1)}, \end{aligned}$$

where $\hat{\mathbf {x}}_1^{(1)}$, $\hat{\mathbf {x}}_2^{(1)}$, and $\hat{\mathbf {x}}_3^{(1)}$ satisfy the following left boundary conditions, respectively:

116 $$\begin{aligned}&\hat{\mathbf {x}}_1^{(1)}(0) =\left( 0,1,0,0,0, \frac{\rho }{\sigma + \rho }, 0, 0, 0, 0\right) ^T,\nonumber \\&\hat{\mathbf {x}}_2^{(1)}(0) =\left( 0,0,1,0,0, 0, 0, 0, 0, \frac{\phi f'(\hat{n}_x^{(0)}(0) - \hat{n}_\tau ^*)}{\sigma }\right) ^T,\nonumber \\&\hat{\mathbf {x}}_2^{(1)}(0) =\left( 0,0,0,0,0, 0, 0, 1, 0, 0\right) ^T. \end{aligned}$$

In other words, the left boundary conditions for $\hat{\mathbf {x}}_1^{(1)}(s)$, $\hat{\mathbf {x}}_2^{(1)}(s)$, and $\hat{\mathbf {x}}_3^{(1)}(s)$ correspond to prescribing $\hat{y}^{(1)}(0) = 1$, $\hat{n}_x^{(1)}(0) = 1$, and $\hat{m}^{(1)}(0) = 1$, respectively. By linear independence, the solution for each copy can be obtained independently, as functions of the eigenvalue, $\sigma$. The constants $c_1$ and $c_2$ and $c_3$ are determined by imposing that the right boundary conditions (112)–(114) are satisfied. This leads to the following matrix equation at $s = l$:

117 $$\begin{aligned} \left( \begin{array}{ccc}\hat{x}_1^{(1)} &{} \hat{x}_2^{(1)} &{} \hat{x}_3^{(1)} \\ \hat{y}_1^{(1)} &{} \hat{y}_2^{(1)} &{} \hat{y}_3^{(1)} \\ {\hat{\theta }}_1^{(1)} &{} {\hat{\theta }}_2^{(1)} &{} {\hat{\theta }}_3^{(1)} \end{array}\right) \left( \begin{array}{c}c_1\\ c_2\\ c_3\end{array}\right) = \left( \begin{array}{c}0\\ 0\\ 0\end{array}\right) , \quad {\text{ at} } \quad s = l. \end{aligned}$$

A solution to (117) exists if and only if the determinant of the matrix on the left-hand side is zero, for a given value of $\sigma$. If all values of $\sigma$ that cause the determinant of (117) to vanish satisfy ${\mathrm {Re}}(\sigma ) < 0$, then the dynamic perturbations decay to zero as $t \rightarrow \infty$. Therefore, the homeostatic solutions are dynamically stable. However, if there is at least one positive value of $\sigma$, then the dynamic perturbations grow exponentially in time and the homeostatic solutions are dynamically unstable.

## Footnotes

1 For notational simplicity, where clear, we will just write $({\mathbf {r}}-{\mathbf {p}})$.

2 The growth stretch of a small section over a time increment $\varDelta t$ is given by $1+{\dot{\gamma }}/\gamma \varDelta t$. Contrast this with the function $\gamma$ itself, which describes the change in arc length at a given material point over the entirety of the growth history.

3 Note that $\frac{ds}{\hat{\alpha}}=dS$, so that (69) is equivalent to integrating the incremental growth over the grown “virtual” configuration.

4 Since we follow only the critical buckling mode, inertial stability is already established for the solutions under consideration.
